# Free-Energy Landscapes of HBV Hexamer Closure Reveal Key Structural Features of the Transition

**DOI:** 10.1021/acs.jctc.6c00439

**Published:** 2026-05-04

**Authors:** Zixing Fan, Anna Pavlova, Diane L. Lynch, Christophe Chipot, James C. Gumbart

**Affiliations:** † Interdisciplinary Bioengineering Graduate Program, 1372 Georgia Institute of Technology, Atlanta, Georgia 30332, United States; ‡ School of Physics, Georgia Institute of Technology, Atlanta, Georgia 30332, United States; § Laboratoire International Associé Centre National de la Recherche Scientifique et University of Illinois at Urbana−Champaign, Unité Mixte de Recherche n°7019, Université de Lorraine, B.P. 70239, Vandœuvre-lès-Nancy cedex 54506, France; ∥ Theoretical and Computational Biophysics Group, NIH Center for Macromolecular Modeling and Visualization, Beckman Institute for Advanced Science and Technology, University of Illinois at Urbana−Champaign, Urbana, Illinois 61801, United States; ⊥ Department of Biochemistry and Molecular Biology, The University of Chicago, Chicago, Illinois 60637, United States; # Department of Chemistry, The University of Hawai’i at M a̅noa, Honolulu, Hawaii 96822, United States; ∇ School of Chemistry & Biochemistry, Georgia Institute of Technology, Atlanta, Georgia 30332, United States

## Abstract

Chronic infection with the hepatitis B virus (HBV) remains a major global health burden and depends on efficient viral replication, including the assembly of the viral capsid during the HBV life cycle. Capsid assembly proceeds through transient oligomeric intermediates, among which the formation of hexameric units is thought to underlie the energetic bottleneck associated with nucleation during capsid assembly. Despite extensive experimental and computational work, the structural and energetic determinants of hexamer closure remain incompletely understood at the molecular level. Here, we employ a multistage computational approach to investigate the open-to-closed transition of the HBV capsid hexamer in the apo system, in which targeted molecular dynamics is employed to generate diverse open-to-closed transition pathways. These pathways are subsequently refined and sampled by using path-based free-energy methods to construct multidimensional free-energy landscapes of hexamer closure. Across independently sampled pathways, we observe diverse transition routes, while a conserved steric rearrangement in the gate region emerges as the dominant rate-limiting feature. These results provide a qualitative characterization of hexamer closure energetics and establish a general framework for studying complex conformational transitions in large, flexible biomolecular assemblies.

## Graphical abstract

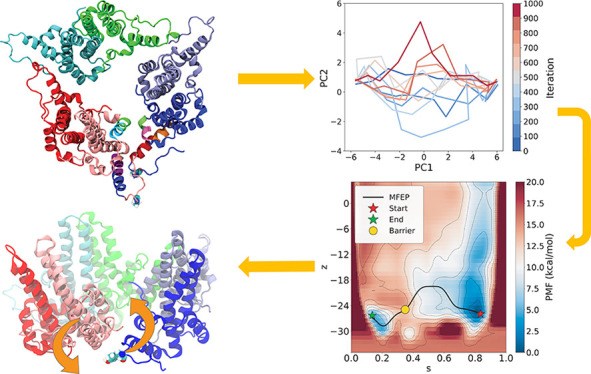

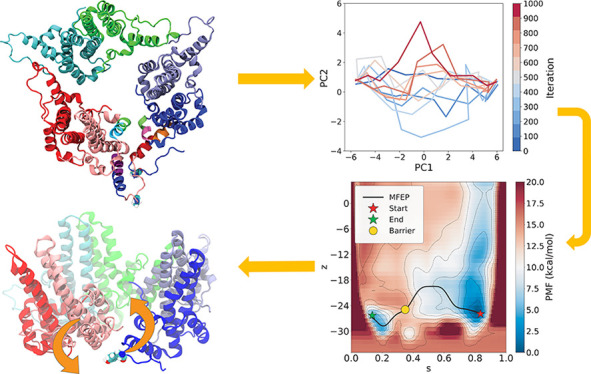

## Introduction

Chronic hepatitis B virus (HBV) infection affects nearly 300 million people worldwide and remains a major cause of liver cancer and cirrhosis, with approximately one million deaths attributed to HBV annually.^1,2^ Although effective vaccines exist, current treatments are limited to interferon-α derivatives and nucleotide analogs, neither of which provides a functional cure and both of which often require long-term administration.^3^ Viral persistence is primarily driven by the formation of covalently closed circular DNA (cccDNA) in the host cell nucleus, which is largely refractory to existing therapies.^3,4^ Consequently, new therapeutic strategies are urgently needed. One promising approach targets the HBV nucleocapsid, an icosahedral protein assembly essential to the viral life cycle: compounds that interfere with capsid assembly have been shown to reduce both viral replication and cccDNA levels.^3,5,6^

HBV capsids assemble from capsid protein (Cp) homodimers, which are the dominant species in solution under low ionic strength conditions.^4,7^ Assembly proceeds spontaneously to form predominantly *T* = 4 icosahedral shells through a series of early oligomeric intermediates, most notably tetramers and hexamers.^8,9^ Numerous experimental and theoretical studies support a nucleation-elongation mechanism in which formation of hexameric units represents a key kinetic bottleneck in capsid assembly.^8,10–12^ Once hexamers are formed, additional dimers or small oligomers are incorporated to complete capsid growth.^8,10,11^ The Cp monomer comprises a 149-residue N-terminal assembly domain and a C-terminal nucleotide-binding region; the isolated assembly domain is sufficient to form capsids nearly identical to those of the full-length protein.^13,14^ This truncated construct therefore provides a well-established model system for probing the molecular mechanisms of the HBV capsid assembly.

Recent small-angle X-ray scattering (SAXS) and time-resolved SAXS studies have further highlighted the importance of early assembly intermediates.^12,15^ Despite the enormous number of theoretically possible intermediates, only a limited subset is sampled under experimentally relevant conditions, with tetramers and hexamers dominating the early stages of assembly.^12,15^ These observations emphasize that capsid assembly is governed by a delicate balance of dimer–dimer interaction energies and suggest that the structural transitions associated with hexamer formation and closure play a central role in controlling the assembly pathways. While nucleation-limited assembly is well established, the structural origin of this barrier during hexamer formation remains elusive.

Small-molecule compounds that interfere with viral capsid assembly, known as capsid assembly modulators (CAMs), have emerged as a promising therapeutic strategy for HBV.^16^ For HBV, most CAMs accelerate capsid assembly, inducing either the formation of empty or aberrant particles, and are commonly classified as either misdirecting or empty-capsid-forming agents.^17^ Despite their structural diversity, CAMs bind at a common site located at the Cp interdimer interface, where they are thought to primarily enhance hydrophobic protein–protein interactions.^18^ Experimental measurements and modeling studies estimate that individual dimer–dimer contacts contribute only a few kcal/mol to capsid stability, and CAM binding further modulates these interactions.^10,19,20^

While experimental and computational studies have begun to reveal how CAMs alter the conformational states of early intermediates and capsid assembly outcomes,^21–25^ key mechanistic aspects remain unresolved. In particular, the pathways and free-energy barriers associated with transitions between early assembly intermediates, especially hexamer closure, are not well characterized at the atomic level. A detailed understanding of competing assembly pathways, metastable intermediates, and kinetic traps will be central to the rational design of CAMs that modulate those pathways in specific ways. The objective of this study is to characterize the molecular mechanism and free-energy landscape of HBV capsid hexamer closure in the absence of CAMs. By focusing on the apo system, we establish a physically grounded baseline for the putative rate-limiting step of capsid assembly and identify the dominant structural bottlenecks governing this transition.

In principle, unbiased MD is capable of exploring conformational state transitions; however, large free-energy barriers make such simulations infeasible due to the typically long time scales of biological processes. Given the importance of delineating transition pathways in the description of thermodynamics and kinetics of biophysical processes, including self-assembly, several alternative path-finding strategies have been developed. Early work focused on guiding the system between states through the application of external forces and includes steered^26^ and targeted MD (SMD and TMD). Furthermore, path sampling approaches have been developed and include transition path sampling (TPS),^27^ a Monte Carlo exploration of the transition path ensemble, and its variants such as transition interface sampling (TIS)^28^ and forward flux sampling (FFS).^29^ Additionally, the weighted ensemble method^30^ and milestoning^31^ produce collections of transition paths by employing the knowledge of a small set of important collective variables (CVs). More recently, machine learning approaches that employ the committor as a CV for enhanced sampling have been proposed.^32,33^ While these methods show considerable promise, their application has thus far been largely focused on smaller systems. More generally, committor-based approaches provide a rigorous framework for defining reaction coordinates for activated transitions and have been used to analyze complex conformational changes in molecular systems.^34–36^

Despite this rigorous foundation, their application to large, highly dimensional biomolecular systems remains challenging. As a result, many studies instead rely on physically motivated collective variables and path-based methods to describe transitions on rugged free-energy landscapes. Within this framework, an attractive option is the string method with swarms of trajectories (SMwST).^37,38^ Here, given starting and ending conformations, the refinement of an approximate transition pathway is determined along a one-dimensional curve, the string, which proceeds from the initial to the final conformational state. Discretization of the string into a collection of images whose positions are iteratively refined yields an optimized transition path. A distinct advantage of string methods is that a collection of CVs is mapped onto the 1D curve, allowing for the introduction of multidimensional CVs without incurring a prohibitive computational cost. Unlike the original CV-based string method,^39^ which is based on a mean-force computation, SMwST relies on a large collection of short trajectories for each image and ensures a zero-drift pathway. With the optimal pathway in hand, an enhanced sampling strategy,^40^ such as metadynamics,^41^ adaptive biasing force,^42^ or their variants, can be performed to compute the corresponding free-energy profile along this pathway. In this manner, computational effort is focused on refining and subsequent sampling of the transition pathway. In fact, SMwST has been applied to a variety of high-dimensional biophysical systems such as G protein-coupled receptors (GPCRs),^43^ membrane transporters,^44^ and ligand-gated ion channels.^45^

Our computational strategy proceeds in several stages. Targeted MD simulations are first used to generate diverse open-to-closed transition trajectories. Representative pathways are then selected and refined using the string method in a 24-dimensional, distance-based CV space describing the interdimer gate region. Free-energy sampling is subsequently performed around each refined pathway using metadynamics-extended adaptive biasing forces (meta-eABF)^46–48^ combined with arithmetic path collective variables, enabling exploration of progress along both the transition coordinate and orthogonal deviations from it. Finally, minimum free-energy paths are extracted from the resulting two-dimensional landscapes to identify the lowest-barrier routes connecting the open and closed hexamer states. This multistage approach enables direct identification of energetic barriers associated with hexamer closure, thereby providing a mechanistic foundation for the nucleation bottleneck observed in the HBV capsid assembly.

Beyond HBV capsid assembly, this work has broader implications for the computational study of large biomolecular systems undergoing complex conformational transitions. Many biologically relevant processes lack a single dominant reaction pathway and instead involve families of related routes separated by modest free-energy differences. By emphasizing reproducible structural bottlenecks rather than unique transition geometries, the workflow presented here provides a practical strategy for extracting robust mechanistic insight from rugged, high-dimensional free-energy landscapes in such systems.

## Results

### Generating Initial Transition Paths with Targeted MD

To generate candidate transition pathways between the open and closed HBV hexamer conformations, we employed TMD simulations connecting the equilibrated open and closed reference structures shown in Figure 1B,C. Three independent TMD trajectories were generated to steer the system from the open toward the closed conformation by using a weak harmonic bias, allowing substantial conformational flexibility and producing multiple realizations of the transition rather than a single prescribed pathway. As a result, the TMD simulations sampled a diverse ensemble of physically plausible transition-like trajectories that capture large-scale structural rearrangements associated with hexamer closure. These trajectories are not interpreted as quantitative transition pathways, but instead serve as biased samples of the conformational space connecting the two end states, thereby providing starting points for subsequent dimensionality reduction and pathway refinement.

**Figure 1:**
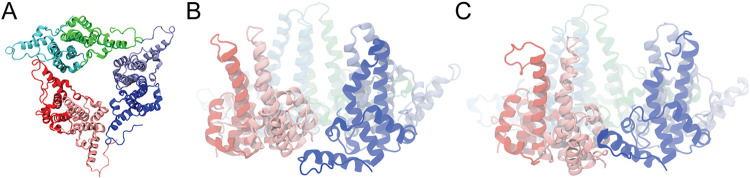
Structural representations of the HBV capsid protein hexamer (Cp149) used in this study. (A) Top view of the Cp149 hexamer, which is a trimer of dimers. (B, C) Side views of the “gate” region in the open (B) and closed (C) conformations.

A comparison of the equilibrated open (Figure 1B) and closed (Figure 1C) hexamer structures indicates that the largest, functionally relevant differences are localized at the interdimer gate region, whereas the remainder of the hexamer undergoes comparatively modest, quasi-rigid rearrangements. We therefore focused our CV description on this gate region. Each interfacial helix was divided into two short segments to define its spatial orientation so that distances between opposing helical segments capture both separation and relative tilt or shear at the interface while remaining insensitive to overall translation and rotation of the hexamer. Within this framework, we defined a set of distance-based CVs consisting of interhelical distances between the selected α-helical segments on chains A and F, which oppose each other across the interface that reorganizes during closure, together with distances involving residue Tyr132 on each chain, a site known to influence stabilization of the closed conformation and subsequent assembly.^49,50^ Distances were computed between the centers of mass of the C_α_ atoms within each helical segment and of Tyr132. Together, these distances provide a high-dimensional representation of the evolving gate-region geometry during hexamer closure and form the basis for subsequent dimensionality reduction and pathway selection (Figure 2). Full definitions of the atomic groups and CVs are provided in Tables S1 and S2.

**Figure 2:**
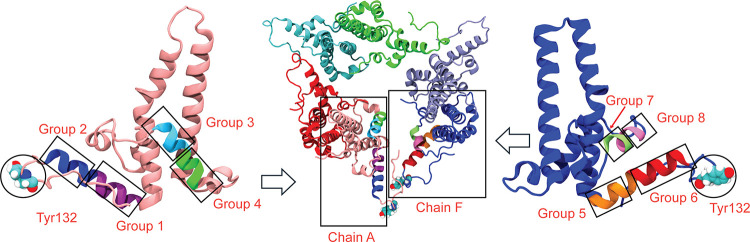
Atomic groups used to construct the 24 distance-based CVs at the hexamer gate region. The central panel shows an overall view of the HBV hexamer, while the left and right panels provide zoomed-in views of the selected regions on chain A and chain F, respectively. Four α-helical segments are defined on chain A (Groups 1–4) and on chain F (Groups 5–8), along with residue Tyr132 on each chain.

### Dimensionality Reduction by Principal Component Analysis and Initial Pathway Identification

To facilitate visualization of the high-dimensional CV space and to identify representative transition pathways for subsequent string-method refinement, we performed principal component analysis (PCA)^51^ on the 24-dimensional CV trajectories obtained by pooling data from all three TMD simulations (Figure 3A). The first two principal components capture a substantial fraction of the total variance, with PC1 accounting for 55% and PC2 accounting for 24%. The coefficients of the individual CVs contributing to these components are summarized in Table S3.

**Figure 3:**
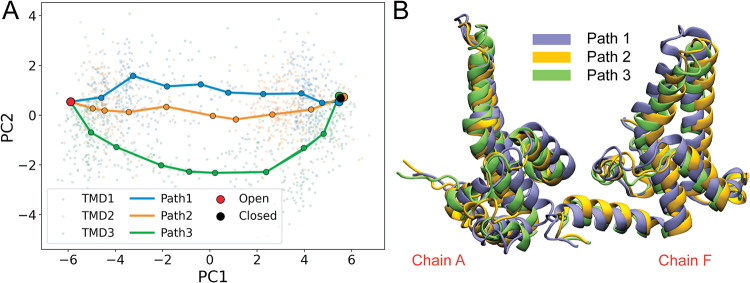
PCA of TMD trajectories and selection of the representative initial pathways. (A) Projection of the three targeted molecular dynamics (TMD) trajectories onto the first two principal components (PC1 and PC2) of the 24-dimensional CV space, with the three representative initial paths selected from these trajectories overlaid. (B) Superposition of representative midpoint structures (image 5) selected from each path highlights pathway-dependent structural differences.

Inspection of the projected trajectories shows that PC1 primarily reflects progress along the open-to-closed transition, with open-like conformations located at negative PC1 values and closed-like conformations at positive values. In contrast, PC2 distinguishes alternative transition routes involving distinct local rearrangements of the interhelical gate region. Although PC2 contributes less variance than PC1, substantial dispersion along this direction indicates significant conformational flexibility in the mechanism by which closure can proceed. The structural interpretation of these principal components, based on distance-based CVs, is illustrated in Figure S1. Consistent with this interpretation, the three independent TMD trajectories occupy overlapping but distinct regions of the PC1-PC2 plane (Figure 3A). While the trajectories largely overlap in the early stages of the transition, they progressively diverge along PC2 as closure proceeds, giving rise to trajectory-specific conformational patterns.

Based on the projection onto PC1 and PC2, we selected ten representative configurations from each TMD trajectory that span the full open-to-closed transition and occupy distinct regions of PC space (Figure 3A). Configurations were selected to span the full range of the transition in the reduced PC space while maintaining approximately uniform spacing in the original CV space, ensuring that each path provided a continuous and representative description of the underlying trajectory. These configurations define the discretized path used for subsequent string-method refinement, with nodes approximately equidistant in the original 24-dimensional CV space to balance structural resolution and computational cost.^37^

To further assess structural differences among the selected pathways, representative midpoint structures (image 5 from each path) were aligned and superimposed (Figure 3B). Although these structures correspond to similar stages along the transition, differences are observed in both the interfacial helices and distal regions of the hexamer, indicating that the closure transition can proceed through multiple structurally distinct pathways rather than following a single dominant trajectory. The complete initial paths, each consisting of ten images, are shown in Figures S2–S4.

### Refinement of Transition Pathways Using the String Method

We refined the three TMD-derived pathways using the string method with swarms of trajectories (SMwST) in the full 24-dimensional distance-based CV space.^37,38^ Each path was represented as a discrete string connecting the open and closed states, with the end points restrained to remain within their respective basins. String refinement was performed iteratively by estimating the local mean drift perpendicular to the current path and updating the image positions accordingly.

To visualize the evolution of the strings during refinement, images from successive iterations were projected onto the first two principal components derived from the CVs (Figure 4A–C). Although all updates were performed in the full 24D space, this projection provides an intuitive representation of how the paths reorganize during refinement. Early iterations are characterized by substantial internal rearrangement of each string as poorly connected or strained segments relax. As the refinement proceeds, the three strings exhibit distinct behaviors. Path 1 spans a broad region of the PC1-PC2 plane, reflecting a highly diverse set of intermediate structures and pronounced pathway dispersion rather than a single dominant transition route. Path 2 also displays significant variability, particularly in later images, which show larger excursions in the reduced space. In contrast, Path 3 progressively organizes into a smooth, well-defined trajectory, with later iterations largely retracing the same route. Notably, this path remains separated from the regions explored by Paths 1 and 2 throughout refinement, indicating a topologically distinct transition pathway within the conformational landscape.

**Figure 4:**
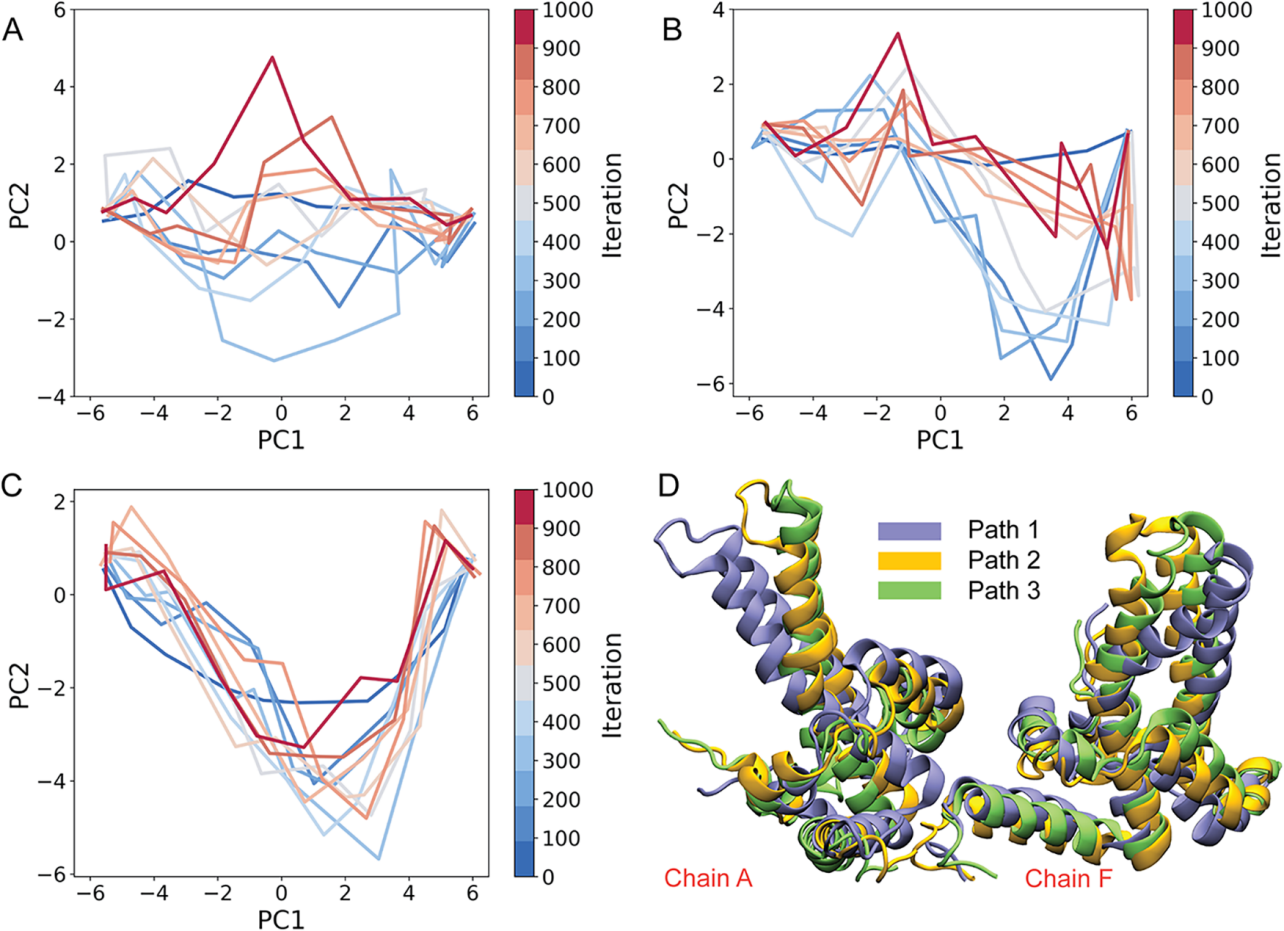
String-method path evolution in a 2D PCA projection and comparison of the refined pathways. (A-C) Paths 1–3 shown in the PC1-PC2 plane across string iterations (0 to 1000 iterations in steps of 100), colored from blue (early) to red (late). (D) Superposition of the refined string midpoint structures (image 5) after 1000 iterations.

Convergence was assessed using the normal root-mean-square deviation (RMSD), which quantifies changes in string shape perpendicular to the instantaneous path. As shown in Figure S5, Path 3 exhibits relatively low and weakly varying normal RMSD values at later iterations, whereas Paths 1 and 2 maintain larger-amplitude fluctuations without a sustained downward trend. These observations are consistent with the behavior seen in the PC1-PC2 projections, where Path 3 adopts a more stationary trajectory (Figure 4C) while Paths 1 and 2 continue to explore a broader region of the reduced space (Figure 4A and B).

Inspection of representative midpoint structures (image 5) shows that the three pathways remain geometrically distinct at intermediate stages of the transition after refinement (Figure 4D). Comparison of the initial and refined strings in the reduced PC space (Figure S6) indicates that, within the sampled number of iterations, the refinement does not lead to collapse onto a single common trajectory. The completely refined strings, represented by ten images per path with corresponding structural conformations of the gate region, are shown in Figures S7–S9. Despite incomplete convergence, string refinement relaxes distortions introduced by TMD initialization and removes poorly connected or highly strained segments, yielding transition routes that are more physically consistent at the local structural level. Given the complexity of the CV space, we retain all three refined strings as candidate pathways for subsequent free-energy sampling. To accommodate residual uncertainty in path definition, subsequent sampling is performed in a two-dimensional path-based coordinate system that explicitly treats progress along the path and orthogonal deviations as coupled degrees of freedom.^52^

### Free-Energy Landscape Sampling Using Arithmetic Path Collective Variables

To quantify the thermodynamics of the open-to-closed transition of the HBV capsid hexamer, we computed two-dimensional free-energy landscapes around each refined string using meta-eABF sampling with arithmetic path collective variables (pathCVs).^52,53^ For a given reference path, the pathCV framework defines two coupled reaction coordinates: a progress coordinate *s*, which parametrizes motion along the reference path, and an orthogonal deviation coordinate *z*, which measures fluctuations transverse to the path. These coordinates enable sampling within a tubular region surrounding the nominal transition route. In this context, the pathCVs serve as practical descriptors for pathway refinement and free-energy sampling rather than as rigorously validated reaction coordinates in the committor sense.

The pathCVs are defined as

1 $$s\left(x\right)=\frac{\sum_{i=1}^{N}i\exp\left[-\lambda d^{2}\left(x,x_{i}\right)\right]}{\sum_{i=1}^{N}\exp\left[-\lambda d^{2}\left(x,x_{i}\right)\right]}$$

2 $$z\left(x\right)=-\frac{1}{\lambda }\ln\left(\sum_{i=1}^{N}\exp\left[-\lambda d^{2}\left(x,x_{i}\right)\right]\right)$$

Here, **x** denotes the instantaneous configuration of the system expressed in the CV space, and **x**_*i*_ represents the configuration corresponding to image *i* along the reference path, with *N* denoting the total number of images defining the reference path. The function *d*(**x**, **x**_*i*_) is the distance between the instantaneous configuration and image *i* in the CV space. The parameter λ is a smoothing parameter that controls the degree of localization of the weighting around nearby path images.

In practice, distances entering the pathCV definitions, *d*(**x**, **x**_*i*_), were evaluated using a subset of 16 CVs selected from the original set of 24 based on their smooth and systematic variation along the refined paths (Figure S10), whereas the full set of 24 CVs was retained during string refinement to preserve structural resolution. Because the pathCV metric is distance-based, inclusion of CVs that exhibit irregular or nonmonotonic behavior along the transition would introduce high-dimensional noise and distort the relative positioning of configurations with respect to the reference path, thereby reducing the stability and interpretability of the resulting free-energy landscapes. For each reference path, extensive meta-eABF sampling was performed in the 2D (*s*,*z*) space, yielding smooth free-energy surfaces *F*(*s*, *z*) that describe both progress along the transition and deviations orthogonal to the reference path. The resulting landscapes are shown in Figure 5A–C.

**Figure 5:**
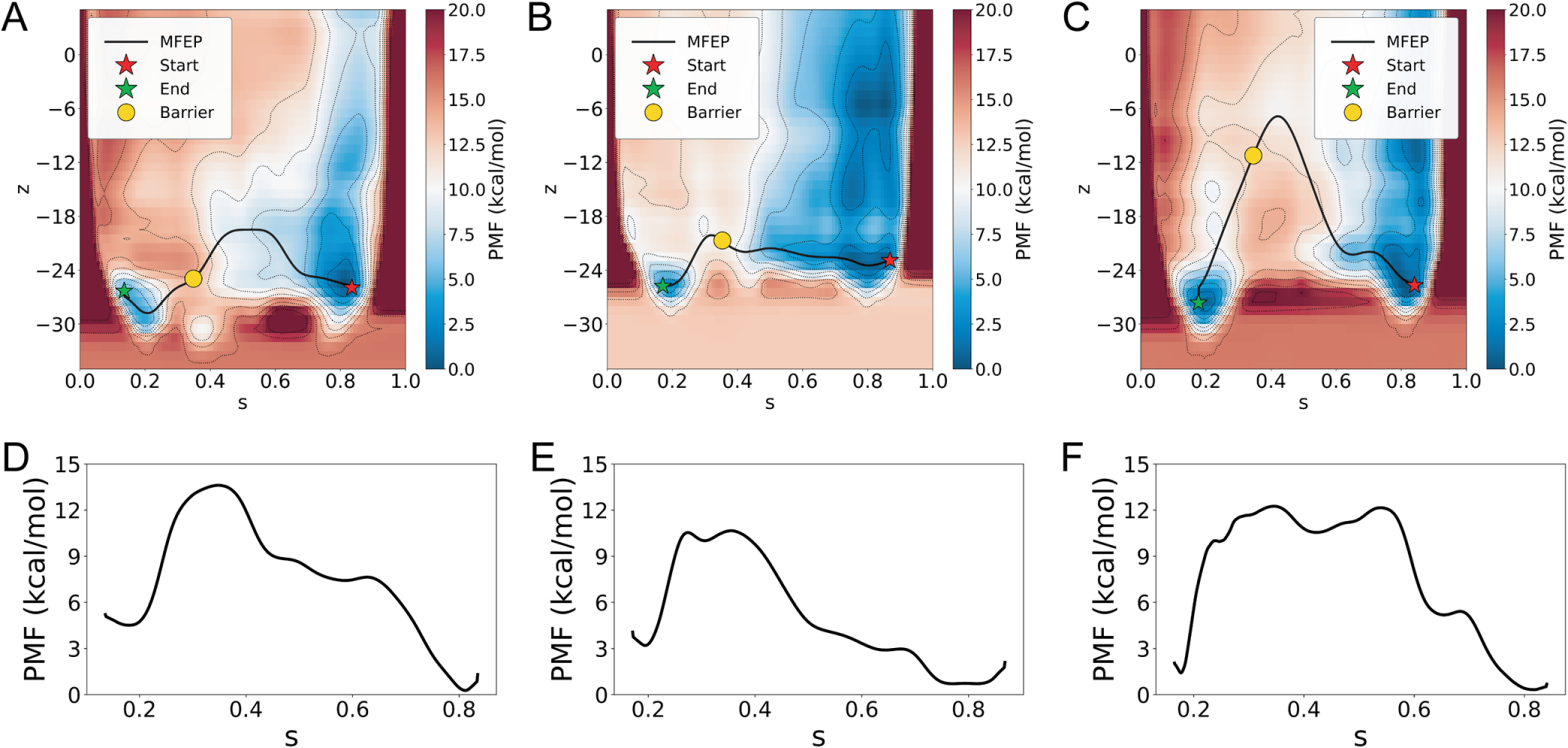
Free-energy landscapes and minimum free-energy paths (MFEPs) for the three refined transition pathways. (A–C) Two-dimensional free-energy surfaces in the arithmetic path CVs (*s*,*z*) constructed around the refined strings, corresponding to Paths 1 through 3, respectively. The open and closed reference conformations are indicated by red and green stars. The MFEP associated with the lowest free-energy barrier on each surface is shown as a black curve, and the corresponding barrier location is marked by a yellow circle. (D–F) One-dimensional PMFs obtained by evaluating the free energy along the MFEP on the two-dimensional surfaces in panels (A–C) as a function of the progress coordinate *s*, corresponding to Paths 1 through 3, respectively.

Across all three landscapes, two well-defined free-energy basins are observed, corresponding to the open and closed hexamer conformations, located near *s* ≈ 0.2 and *s* ≈ 0.8, respectively (Figure 5A–C). In each case, the closed basin lies slightly lower in free energy, suggesting that closed HBV hexamers are modestly more stable than their open counterparts. The open basin is relatively narrow, whereas the closed basin spans a broader region of the (*s*,*z*) space, indicating greater configurational diversity near the closed state at comparable values of *s*.^24^ In all three landscapes, the two basins are separated by a single dominant free-energy barrier observed near *s* ≈ 0.4, suggesting that the principal energetic cost of closure is incurred at an early stage of the transition.

### Minimum Free-Energy Path Identification on the PathCV Landscapes

To identify transition pathways on the computed free-energy surfaces, minimum free-energy paths (MFEPs) were extracted directly at the landscape level. For each two-dimensional (*s*,*z*) free-energy surface, candidate paths connecting the open and closed basins were identified on the discretized surface by using a minimax optimization procedure. The primary objective was to minimize the maximum free energy encountered along the path, corresponding to the identification of the lowest-barrier route between the two basins.^54^ The resulting MFEPs are shown in Figure 5A–C, with the open and closed end points marked by red and green stars, respectively. In all three landscapes, the MFEPs connect the two basins through a single dominant free-energy barrier, indicated by a yellow circle. One-dimensional free-energy profiles obtained by evaluating the free energy along each MFEP on the corresponding two-dimensional surface are shown in Figure 5D–F.

Table S4 summarizes the key thermodynamic quantities extracted from the MFEPs, including the free-energy difference between the open and closed basins and the corresponding barrier heights relative to the open state. The start and end free energies are obtained from the one-dimensional PMF evaluated along each MFEP and are defined as local minima in the vicinity of the open and closed basins, respectively. Across the three pathways, the closed hexamer is modestly favored with an average free-energy difference of −2.6 ± 1.6 kcal/mol. This small energy difference is broadly consistent with previous structural analyses, suggesting that the open and closed hexamer conformations are close in free energy.^49^ In addition, each MFEP has a single dominant barrier with an average barrier height of 9.1 ± 1.7 kcal/mol. While the precise barrier heights and basin offsets vary among the paths, these differences likely reflect residual sampling uncertainty and the high dimensionality of the CV space. Although hexamer formation has been proposed as a rate-limiting step in capsid assembly,^8,10–12^ its mechanistic origin remains unclear. Our results reveal a distinct free-energy barrier for hexamer closure, providing a plausible energetic basis for the slowed kinetics of this process.

Because the MFEPs were extracted from free-energy surfaces constructed in a reduced collective-variable space and obtained from finite sampling, the absolute free-energy values and detailed pathway geometries should be interpreted with appropriate caution. In addition, the incomplete convergence of the refined strings introduces residual uncertainty into the path description. Nevertheless, the qualitative features of the landscapes are consistent across the independently sampled pathways: in all cases, the free-energy surfaces exhibit two well-defined basins connected by a single dominant barrier with the closed state modestly favored relative to the open state. These reproducible features support a robust qualitative mechanistic picture, even though some uncertainty in the precise barrier heights and path geometries remains.

### Realization of Minimum Free-Energy Paths and Identification of the Rate-Limiting Configuration

To relate the bin-level MFEPs extracted from the free-energy landscapes to physically meaningful molecular transitions, we realized these paths in the underlying CV space. Because the free-energy surfaces are defined in a reduced 2D representation, each bin along an MFEP corresponds to a broad ensemble of configurations in the higher-dimensional space. Representative molecular pathways were therefore constructed by selecting configurations from the sampled trajectories associated with each bin along the MFEP such that successive configurations remain close in the original high-dimensional CV space, yielding a continuous molecular progression between neighboring bins. The resulting realized pathways, projected onto the reduced PC space, are shown in Figure S11.

Although the free-energy sampling for all three systems was initiated from refined string references (Figure S6B), the realized MFEPs do not necessarily remain close to their respective reference paths, reflecting some residual uncertainty in the refined strings. Paths 1 and 2 remain broadly consistent with their refined strings, with Path 1 occupying higher PC2 values and Path 2 sampling an intermediate region. In contrast, Path 3 undergoes a substantially greater reorganization. While its refined string spans lower PC2 values (Figure 4C), the corresponding MFEP traverses regions of relatively large orthogonal deviation *z* (Figure 5C), indicating a pronounced departure from the reference path during free-energy optimization. Consequently, the final realized pathway associated with Path 3 converges toward a trajectory structurally similar to Path 2, despite their distinct initial string geometries.

The free-energy barriers along the realized pathways are highlighted in Figure S11 by yellow circles. In all three cases, the dominant barrier is observed at similar values of PC1, near PC1 ≈ 0.4, suggesting that the rate-limiting event takes place at an early stage of the open-to-closed transition. To assess the structural similarity of the barrier states, the corresponding molecular conformations extracted from the three realized pathways were overlaid, as shown in Figure 6A. These structures exhibit substantial overlap, indicating that despite following distinct transition routes at later stages, all pathways pass through closely related configurations at this stage of the closure process. Animations of the full transitions along each realized pathway are provided as Supporting Movies 1, 2, and 3.

**Figure 6:**
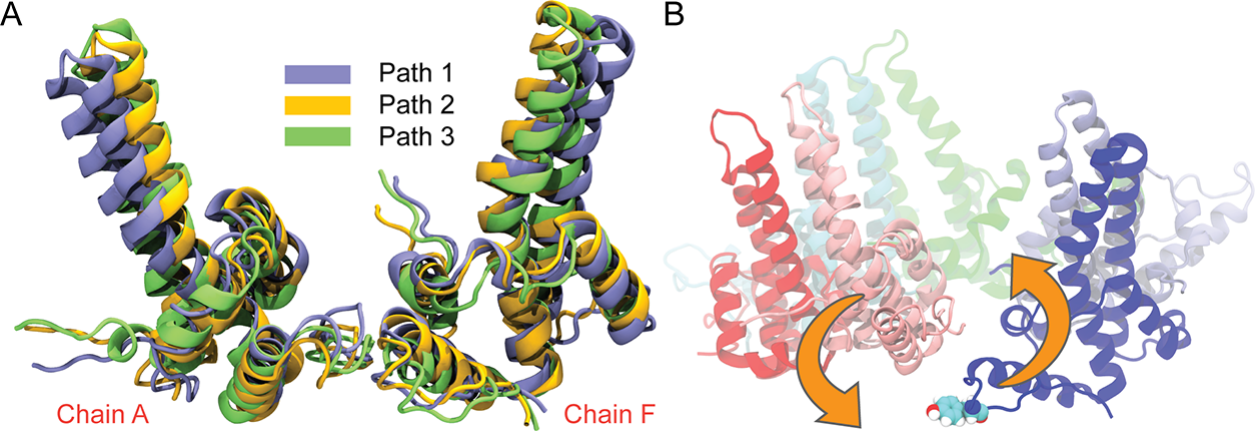
Rate-limiting configuration and illustration of the hexamer closure transition. (A) Structural overlay of the barrier configurations extracted from the three realized MFEPs. (B) Illustration of the hexamer closure transition. Tyr132 of chain F is highlighted in a van der Waals representation. The orange arrows illustrate the transition from open to closed conformation.

### Structural Origin of the Free-Energy Barrier Involving Tyr132

Comparison of the barrier structures from Path 2 (Figure 7B) with the representative open and closed conformations (Figure 7A,C) provides structural insight into the origin of the free-energy barrier associated with hexamer closure. In the open conformation, the helices of chain F (blue) lie below those of chain A (pink), whereas in the closed conformation, the helices of chain F are positioned above those of chain A. In both end states, the two chains remain vertically separated, allowing them to accommodate one another without significant steric conflict. In contrast, at the rate-limiting configurations (Figure 7B), the helices of the two chains are forced to pass past one another as chain F transitions from below to above chain A, as illustrated in Figure 6B. This rearrangement is accompanied by the motion of the C-terminal loop of chain A, which must also pass past the narrow interhelical region during the transition. As a result, both the helix and the C-terminal loop of chain A experience pronounced steric confinement as chain F moves upward.

**Figure 7:**
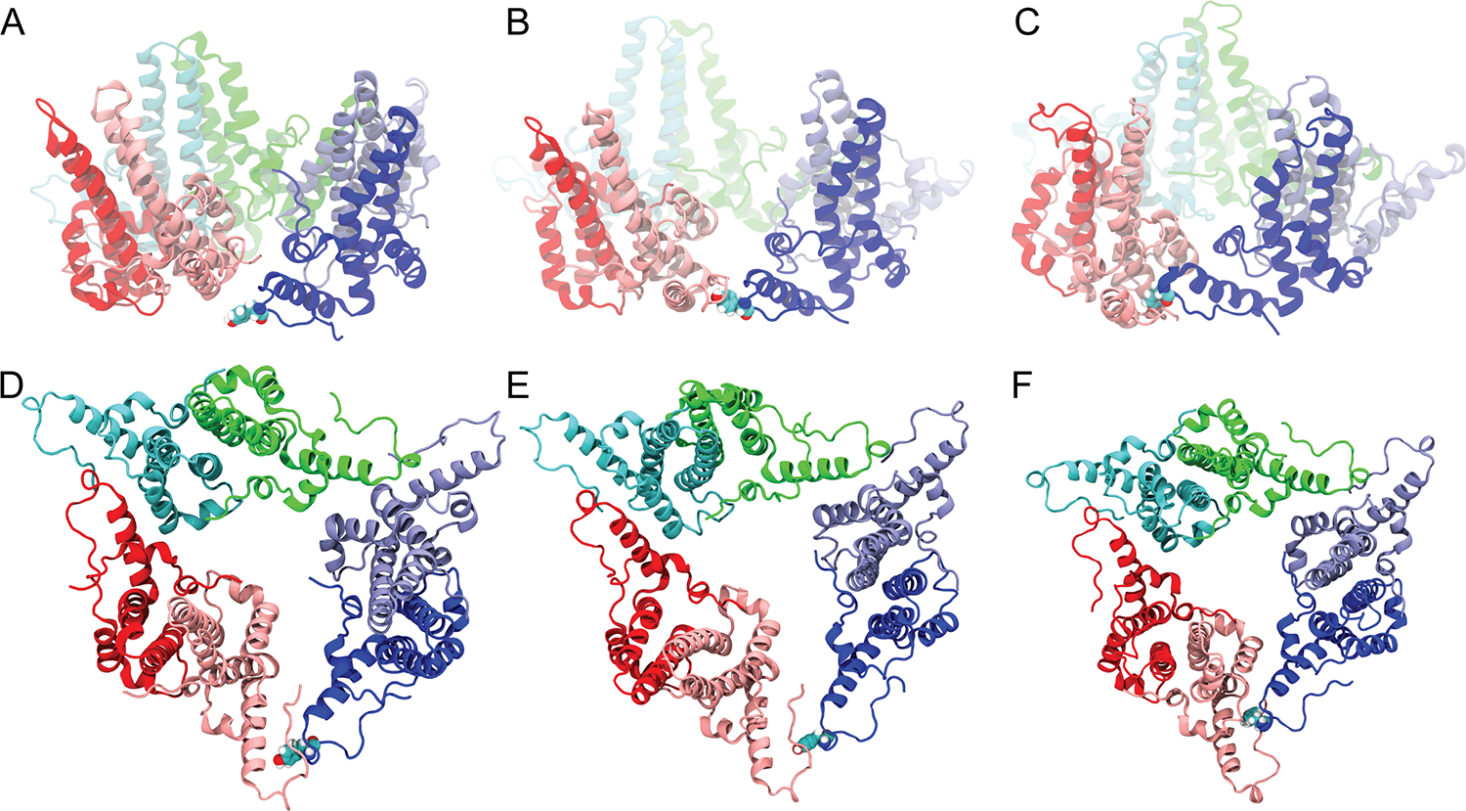
Comparison of representative conformations along the HBV hexamer closure pathway. (A–C) Side views of the hexamer in the open (A), barrier (B), and closed (C) states. (D–F) Corresponding top views of the open (D), barrier (E), and closed (F) states. Tyr132 of chain F is highlighted in a van der Waals representation.

A key contributor to this steric hindrance is Tyr132 of chain F, a bulky residue that points toward chain A during the transition. In the representative barrier configuration (Figure 7B), Tyr132 is in close contact with the C-terminal loop of chain A, suggesting that steric interactions involving this residue may contribute significantly to the observed free-energy barrier, as inferred from the structural comparison of these configurations. To accommodate this clash, the hexamer undergoes a concerted expansion at the barrier conformation, as seen in the top views (Figure 7D–F), indicating that local steric frustration propagates into a global deformation of the assembly.

Once the closure is complete, Tyr132 occupies a position that stabilizes the closed geometry and disfavors reopening by reintroducing steric constraints that oppose the reverse motion (Figure 7F). This interpretation is consistent with experimental studies showing that the Y132A mutation alters hexamer closure behavior: replacement of tyrosine with the less bulky alanine is expected to reduce steric hindrance during both closing and reopening. Given the modest free-energy difference between the open and closed states observed here, such a reduction in steric stabilization would be expected to facilitate reopening of the closed hexamer, in agreement with experimental observations that the Y132A mutant displays an unstable closed hexamer and defective assembly.^49,50^

## Discussion

By examining hexamer closure using a high-dimensional, path-based free-energy framework, we find that the transition exhibits substantial pathway variability while being constrained by a conserved steric barrier at the interdimer gate region. This work has important implications for both free-energy sampling of complicated systems and understanding the structural basis of HBV capsid assembly. From a methodological perspective, the multistage workflow employed here demonstrates a practical strategy for resolving conformational transitions in large, flexible protein assemblies by combining flexible steering, pathway refinement, and path-based free-energy sampling. From a mechanistic perspective, the resulting free-energy landscapes provide a framework for interpreting how specific conformational rearrangements within the hexamer shape the energetic bottlenecks associated with the early stages of capsid assembly.

When this approach is applied to large, highly flexible systems such as the HBV capsid hexamer, several practical considerations emerge. During initial pathway generation, it is essential to sample structurally diverse transition trajectories rather than a single prescribed route. To this end, we employed targeted MD with a relatively weak steering bias, allowing the system to explore its intrinsic conformational flexibility while still being guided toward the target state.^55^ This strategy avoids a purely linear interpolation between end points and enables the generation of transition-like trajectories that better reflect the intrinsic flexibility of the hexamer. Maintaining this diversity through subsequent refinement and free-energy sampling proved to be essential.^37^ By explicitly modeling three distinct initial paths, we were able to assess the robustness of the inferred mechanisms and distinguish pathway-dependent features from shared energetic constraints. During subsequent free-energy sampling, deviations from the refined strings reflect the fact that the string provides only an approximate representation of the transition pathway in the reduced CV space. Consistent with this, although the refined strings remained substantially different from one another, two of the resulting pathways showed partial convergence during free-energy sampling and path extraction, and all three exhibited a closely related dominant free-energy barrier. Among these pathways, Path 3 remained confined to low PC2 values throughout string refinement (Figure 4C), indicating that local features of the reduced landscape can influence refinement outcomes in a large and flexible system such as the HBV hexamer. This interpretation is supported by an alternative path search for Path 3 constrained to remain near the refined string, which yielded a higher barrier and a suboptimal pathway (Figure S12). Together, these observations demonstrate that sampling multiple pathways is necessary not only for consistency checks but also to identify and avoid locally optimal yet globally suboptimal transition routes.

Beyond the initial generation of diverse pathways, the behavior observed during string refinement underscores the importance of monitoring convergence. Although convergence is often readily achieved in simpler systems within a finite number of iterations,^37^ such behavior is not guaranteed in more complex systems. In our system, the most substantial improvements to the initial paths occurred during the early stages of refinement, when poorly connected or highly strained segments were relaxed (Figure S5). Beyond several hundred iterations, the strings largely exhibited fluctuations without clear convergence toward a unique pathway. This behavior likely reflects the absence of a single, well-defined minimum free-energy transition route for the HBV hexamer. For such systems, continued refinement without careful monitoring of string stability can be misleading,^56^ and stopping early combined with downstream free-energy sampling may provide a more informative description than running longer refinements.

In addition, the selection and dimensionality of CVs play a central role in both string refinement and free-energy sampling.^37^ In this study, we focused on distance-based CVs describing the gate region using 24 variables for string refinement and a reduced set of 16 for pathCV sampling. Including additional CVs can capture more structural detail but at the cost of increased complexity and uncertainty, particularly because each bin in the reduced pathCV space corresponds to a large ensemble of configurations in the underlying high-dimensional space.^57^ Moreover, because pathCVs rely on distances in CV space, the inclusion of variables that fluctuate independently and do not vary systematically along the transition can introduce high-dimensional noise into the path definition. The observed consistency of the barrier conformations at the gate region across all three pathways suggests that the chosen variables capture a key energetic feature of the hexamer closure transition. At the same time, examination of the full hexamer beyond the gate region (Figure S13) reveals substantial structural variability in other parts of the assembly. Although these motions were not explicitly included in the CV definition, they may contribute indirectly to the variance in the computed free-energy values. This highlights the practical trade-off between locality and completeness in CV design: focusing on a well-defined functional region enables clearer mechanistic interpretation, while residual degrees of freedom elsewhere in the system can still influence quantitative estimates.^58,59^

Beyond these methodological considerations, analysis of the barrier conformations provides direct insight into the physical origin of HBV hexamer closure and its potential functional implications. The barrier state corresponds to a transient configuration in which steric congestion at the gate region is maximized as helices and loops from neighboring chains are forced to pass through one another during the transition. In particular, the bulky Tyr132 residue of chain F is prominently involved in this process by coming into close contact with the C-terminal loop of chain A, generating steric repulsion that contributes substantially to the free-energy barrier. To accommodate this unfavorable interaction, the hexamer undergoes a concerted expansion, demonstrating how local steric interactions propagate into a global deformation of the assembly. Once closure is completed, the positioning of Tyr132 also stabilizes the closed geometry, consistent with experimental observations that mutations at this site alter the hexamer stability and assembly behavior. These results imply that mutations reducing steric bulk at Tyr132, such as Y132A, are expected to lower the effective barrier to closure and alter the balance between open and closed hexamer conformations, which could be probed experimentally through changes in the assembly kinetics or shifts in population distributions. Despite these mechanistic insights, the detailed pathway geometry and barrier heights remain subject to uncertainty due to finite sampling and incomplete path convergence. Nevertheless, the consistent qualitative features observed across independently determined pathways support the robustness of the mechanistic interpretation presented here. While local features of the transition may depend on the specific choice of end-point conformations, the dominant early barrier identified here is consistently observed across all sampled pathways.

The apo free-energy landscape established here provides a baseline for understanding how CAMs and mutations may perturb hexamer closure. In particular, the identification of a conserved early barrier associated with steric interactions at the gate region suggests that CAM binding at the interdimer interface may influence assembly kinetics by stabilizing or destabilizing conformations on either side of the barrier. Similarly, mutations that alter the steric environment of the gate region, including at Tyr132 or neighboring residues, may shift the barrier height and thereby influence the nucleation efficiency. Together, these observations provide a structural and energetic framework for interpreting how chemical or sequence perturbations may affect HBV capsid assembly pathways.

In total, our results provide a structural explanation for the rate-limiting nature of hexamer formation in the HBV capsid assembly. Although nucleation–elongation kinetics have been established through experimental approaches such as X-ray scattering and size-exclusion chromatography and supported by higher-level computational models, the molecular origin of the nucleation barrier has remained unresolved. By constructing multidimensional free-energy landscapes at atomic resolution, we identify steric interactions that are responsible for the dominant free-energy barrier to hexamer closure. In this way, our study establishes an atomistic foundation for the nucleation bottleneck and directly links established kinetic models to a specific structural mechanism.

## Conclusions

In this work, we combined TMD, string-method refinement, and path-based free-energy sampling to characterize the open-to-closed transition of the HBV capsid hexamer. By explicitly sampling multiple candidate pathways and realizing them in molecular space, we show that distinct transition routes nevertheless funnel through a common rate-limiting conformation localized in the gate region. Our results highlight both the strengths and limitations of reduced CV descriptions for large assemblies and underscore the importance of multipath sampling when no single dominant transition route exists. More broadly, this framework provides a practical strategy for dissecting conformational transitions in complex multimeric systems, where flexibility, degeneracy, and local bottlenecks coexist.

## Methods

### Molecular Dynamics

All molecular dynamics simulations were performed using NAMD3.^60^ The CHARMM36m force field was used for proteins, while CHARMM36 parameters were used for ions and TIP3P for water.^61,62^ Simulations were carried out on HBV capsid hexamers in either the apo open or closed conformation. The closed-state hexamer structure was taken from PDB entry 4BMG.^63^ The open-state hexamer was based on PDB entry 3KXS and modified to restore the wild-type tyrosine at position 132.^49^ No ligands or CAMs were included in any simulation. Each system was solvated in explicit water under periodic boundary conditions and ionized to a physiological salt concentration of 0.15 M NaCl. The resulting systems contained approximately 130,000 atoms.

Temperature was maintained at 310 K using Langevin dynamics with a damping coefficient of 1.0 ps^–1^. Pressure was maintained at 1 atm using anisotropic Langevin piston pressure coupling. All covalent bonds involving hydrogen atoms were constrained, allowing a 2 fs integration time step.^64^ At each time step, bonded interactions and short-range nonbonded interactions were evaluated within a 12-Å cutoff with a smooth switching function applied between 10 and 12 Å. Long-range electrostatic interactions were treated using the particle-mesh Ewald method and updated every time step.^65^ All systems were energy minimized and equilibrated for 10 ns prior to the TMD simulations. All systems were energy minimized and equilibrated using a multi-step protocol. Position restraints were applied to all protein atoms for 1 ns, followed by 10 ns of equilibration with backbone restraints. This was followed by 10 ns of unrestrained equilibration prior to production simulations. Trajectory visualization and analysis were performed using VMD.^66^

### Targeted Molecular Dynamics Simulations

TMD simulations were used to generate initial transition trajectories between the open and closed HBV hexamer conformations. Three independent TMD simulations were performed, each initiated from the equilibrated open-state structure and steered toward the closed-state reference structure. In each simulation, a harmonic restraint was applied to the backbone atoms to gradually reduce the RMSD to the target structure. A relatively weak force constant of 0.5 kcal mol^–1^ Å^–2^ was used to preserve the conformational flexibility during the transition. Each TMD simulation was run for 100 ns, yielding three candidate transition trajectories connecting the two end states. The resulting trajectories were used to identify representative transition pathways that span the full open-to-closed conformational change and serve as initial inputs for subsequent string-method refinement.

### Principal Component Analysis

To describe structural changes during hexamer closure, a set of distance-based CVs were defined to capture rearrangements in the interdimer gate region. The CVs were constructed from interhelical distances between selected α-helical segments on chains A and F, which face each other across the gate interface, together with distances involving residue Tyr132 on each chain. In total, 24 CVs were defined as distances between centers of mass calculated over C_α_ atoms, providing a 24-dimensional representation of the gate-region geometry. Full definitions of the atomic groups and CVs are provided in Tables S1 and S2.

PCA was performed on the CV trajectories obtained from the TMD simulations to obtain a reduced representation of the conformational space. Data from all three independent TMD trials were pooled prior to PCA to ensure a common basis for comparison across trajectories. Configurations were saved every 0.2 ns from each of the three 100 ns TMD trajectories, yielding a total of 1500 configurations for PCA. Prior to PCA, the CVs were standardized to zero mean and unit variance. The first two principal components were retained for analysis and visualization.

### String Calculations

Transition pathway refinement was performed using the string method with swarms of trajectories in the full 24-dimensional CV space.^37,38^ Three initial paths obtained from the TMD simulations were used as starting guesses. These paths were constructed by selecting configurations that are well separated in the reduced PC space to capture distinct regions of the transition. The selected configurations were then evaluated in the full 24-dimensional CV space and iteratively refined to ensure approximately uniform spacing between successive images, such that no segment length differed by more than a factor of 2 from its neighboring segments. This procedure yields continuous paths while preserving separation in the PC space. The resulting configurations define discretized paths consisting of ten images, including the open and closed end points. The end-point images were restrained throughout the refinement to remain within their respective basins using harmonic restraints applied in CV space with a force constant of 0.5 kcal mol^–1^ Å^–2^. All intermediate images were free to evolve under the string updates, subject only to postupdate equilibration restraints used to recenter the bias.

At each string iteration, the local mean drift for each image was estimated by launching a swarm of 20 short unbiased trajectories. Each swarm trajectory was propagated for 50 MD steps (100 fs) at 310 K. Image updates were performed by using only the component of the mean drift perpendicular to the instantaneous string. After each update, the string was smoothed using a smoothing parameter of 0.2 and reparameterized to maintain approximately uniform spacing between images in the CV space.

Following each string update, all images were equilibrated under harmonic restraints in CV space for 50,000 MD steps (100 ps) to recenter the bias at the updated image positions, using a force constant of 0.5 kcal mol^–1^ Å^–2^. String refinement was carried out for a total of 1000 iterations per path with configurations saved every 100 iterations for analysis. Convergence was monitored by using the normal RMSD between successive string iterations. For each image, the displacement vector between positions at consecutive iterations was computed in the full CV space and the component parallel to the local string tangent was removed. The normal RMSD was calculated as the root-mean-square of the remaining perpendicular displacements, averaged over all images along the string.

### Path Collective Variables and Meta-eABF Free-Energy Sampling

Free-energy sampling was performed using the meta-eABF method as implemented in the Colvars module of NAMD.^46–48,53^ Sampling was carried out along the arithmetic path collective variables (pathCVs) constructed from the refined string pathways.^52,53^ Separate simulations were performed for each of the three refined paths.

For each reference path, two pathCVs were defined: a progress coordinate *s* (eq 1), which parametrizes advancement along the reference path, and an orthogonal deviation coordinate *z* (eq 2), which quantifies deviation transverse to the path. Each reference path consisted of 10 discrete images obtained from string refinement and expressed in CV space. Distances entering the pathCV definitions were evaluated using a reduced subset of 16 distance-based CVs selected from the original set of 24 based on their smooth and systematic variation along the refined paths. This reduction affected only the construction of the pathCV distance metric; all atomic degrees of freedom and all original CVs remained unconstrained during sampling.

The pathCVs were constructed using a smooth weighting scheme over the reference images, controlled by the localization parameter λ. This parameter was chosen to be comparable to the inverse of the mean square displacement between successive configurations along the reference path, ensuring smooth interpolation between neighboring images and a continuous increase of the progress coordinate *s* along the path without discontinuities.^52^

During meta-eABF sampling, the coordinates *s* and *z* were confined using harmonic boundary potentials of 1 kcal mol^–1^ Å^–2^ to restrict sampling to a finite region of the pathCV space. The progress coordinate *s* was confined to the range [−0.1, 1.1] with a grid spacing of 0.01, while the deviation coordinate *z* was confined to the range [−50, 50] with a grid spacing of 1.0. Boundary expansion was enabled to avoid artificial confinement at the edges of the sampled region.

Meta-eABF sampling was carried out in the two-dimensional (*s*,*z*) space.^46^ For the ABF component, mean forces were accumulated on a regular grid and biasing was applied once a minimum of 2000 samples per grid bin had been collected. Metadynamics biasing was applied concurrently along both *s* and *z* using a well-tempered scheme. Gaussian hills with a width of 8.0 (in the dimensionless pathCV coordinates) and an initial height of 0.2 kcal/mol were deposited at a bias temperature of 4000 K. Biasing histories were recorded throughout the simulations.

All simulations were performed at 310 K, and each system was sampled for at least 8 μs of aggregate simulation time per path. Mean forces accumulated during sampling were integrated using a spline-based quadrature to obtain smooth two-dimensional free-energy surfaces *F*(*s*, *z*). Prior to force integration, the *s* and *z* coordinates were rescaled to equalize their variances and a weak Gaussian regularization was applied to suppress numerical noise.

### Minimum Free-Energy Path Identification on Discretized Free-Energy Surfaces

Minimum free-energy paths (MFEPs) were identified directly on discretized 2D free-energy surfaces *F*(*s*, *z*) obtained from meta-eABF sampling. The PMF grid was treated as a graph whose nodes correspond to PMF bins and whose node weights are the associated free energies. The start and end nodes were defined by mapping representative open and closed conformations, selected from low free energy basins of the PMF, to their corresponding PMF bins while ensuring geometric consistency with the respective string reference end points.

Bin-level MFEP candidates were identified directly on the discretized two-dimensional free-energy surface. The primary optimization objective was to minimize the maximum free energy encountered along the path (minimax criterion), corresponding to the identification of a minimum-barrier pathway between the two basins. This minimax optimization was solved using dynamic programming on a directed acyclic graph constrained to forward progress along the path coordinate *s*. Among paths sharing the same minimum barrier, ties were resolved by minimizing the mean free energy along the path.

### Realization of MFEPs as Coherent Molecular Pathways

To obtain physically meaningful transition pathways, MFEPs were realized as discrete sequences of molecular dynamics frames that reside in successive MFEP bins and remain locally coherent in CV space.^52^ Trajectory frames were collected from the reweighted meta-eABF simulations across all sampling windows and iterations and assigned to PMF bins according to their instantaneous (*s*,*z*) values. Path realization was performed using a beam-search procedure that constructs a continuous sequence of frames consistent with the bin-level MFEP. At each step, candidate frames in the next MFEP bin were selected based on proximity in standardized CV space, ensuring local geometric continuity along the pathway. The resulting MFEP realization is a discrete, ordered sequence of molecular configurations, one per MFEP bin, that is continuous in CV space and explicitly anchored to physically representative open and closed end-point structures.

## Supplementary Material









## Data Availability

The data set can be accessed via DOI: 10.5281/zenodo.18894347. The repository contains representative input files for the string method calculations and PathCV sampling simulations, as well as the resulting optimized string and path structures.

## Notes

Additional figures and analysis, including convergence data and intermediate structures, as well as movies of the optimized paths (PDF)
Path 1 MFEP (Movie S1) (MP4)
Path 2 MFEP (Movie S2) (MP4)
Path 3 MFEP (Movie S3) (MP4)
