# Supplementary material for: Free-Energy Landscapes of HBV Hexamer Closure Reveal Key Structural Features of the Transition
Source: J Chem Theory Comput. 2026 May 4;22(10):5348–60. doi: 10.1021/acs.jctc.6c00439 (PMC13217544; doi:10.1021/acs.jctc.6c00439)
Supplement: Supplementary file 1 [file ct6c00439_si_001.pdf]

Supporting Information
Free-Energy Landscapes of HBV Hexamer Closure Reveal Key Structural Features of the Transition

Zixing Fan,[†] Anna Pavlova,[‡] Diane L. Lynch,[‡] Christophe Chipot,[¶,§,‖,⊥] and James C. Gumbart*,[‡,#]

†*Interdisciplinary Bioengineering Graduate Program, Georgia Institute of Technology, Atlanta, Georgia 30332, United States*

‡*School of Physics, Georgia Institute of Technology, Atlanta, Georgia 30332, United States*

¶*Laboratoire International Associé Centre National de la Recherche Scientifique et University of Illinois at Urbana-Champaign, Unité Mixte de Recherche n°7019, Université de Lorraine, B.P. 70239, Vandœuvre-lès-Nancy cedex 54506, France*

§*Theoretical and Computational Biophysics Group, NIH Center for Macromolecular Modeling and Visualization, Beckman Institute for Advanced Science and Technology, University of Illinois at Urbana-Champaign, Urbana, Illinois 61801, United States*

‖*Department of Biochemistry and Molecular Biology, The University of Chicago, Chicago, Illinois 60637, United States*

⊥*Department of Chemistry, The University of Hawai'i at Mānoa, Honolulu, Hawaii 96822, United States*

#*School of Chemistry & Biochemistry, Georgia Institute of Technology, Atlanta, Georgia 30332, United States*

* E-mail: gumbart@physics.gatech.edu

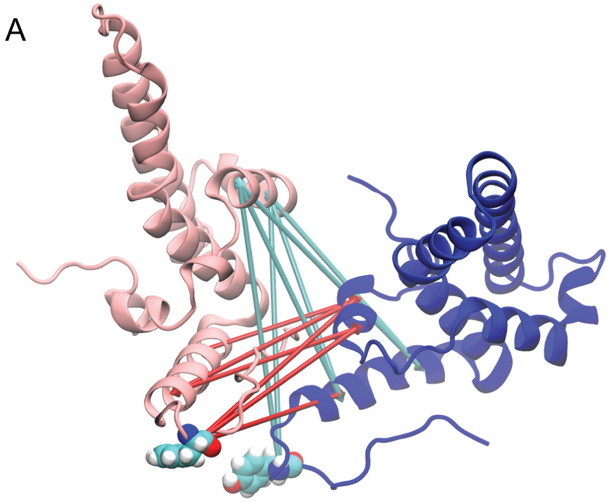

Figure S1: Structural visualization of dominant distance-based collective variables contributing to the first two principal components. Double-headed arrows schematically represent distance collective variables defined between group centroids on chains A (pink) and F (blue); arrow color indicates the sign of the PCA loading (red, positive; cyan, negative). Collective variable definitions correspond to those shown in Fig. 2 and detailed in Tables S1 and S2. **(A)** Collective variables contributing most strongly to PC1. **(B)** Collective variables contributing most strongly to PC2.

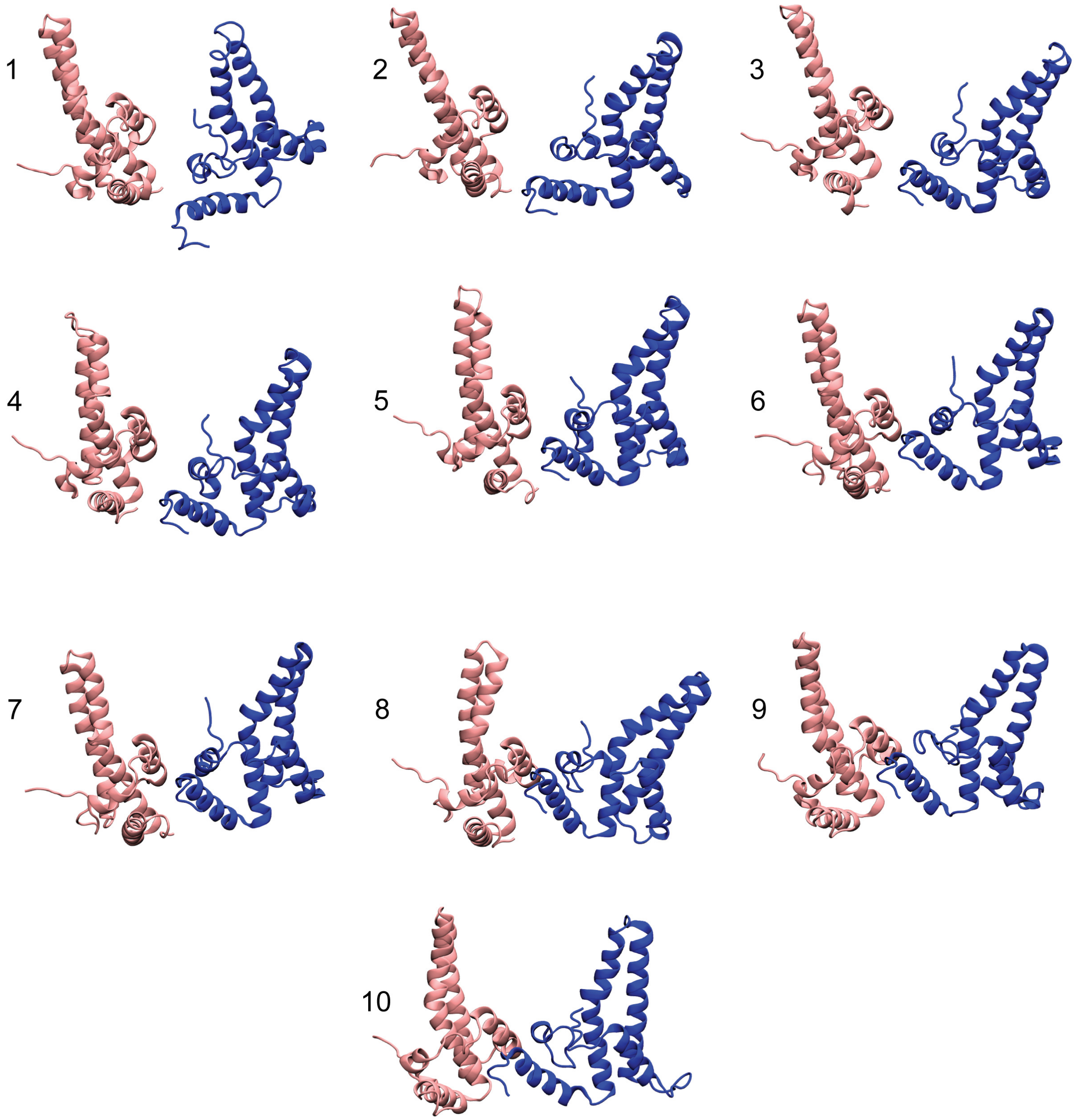

Figure S2: Representative structures along the initial Path 1 selected from targeted molecular dynamics simulations. Images 1–10 correspond to the ten nodes extracted from a single TMD 1 trajectory and used to initialize subsequent string refinement. Structures are shown with a focused view of the inter-dimer gate region involving chains A and F.

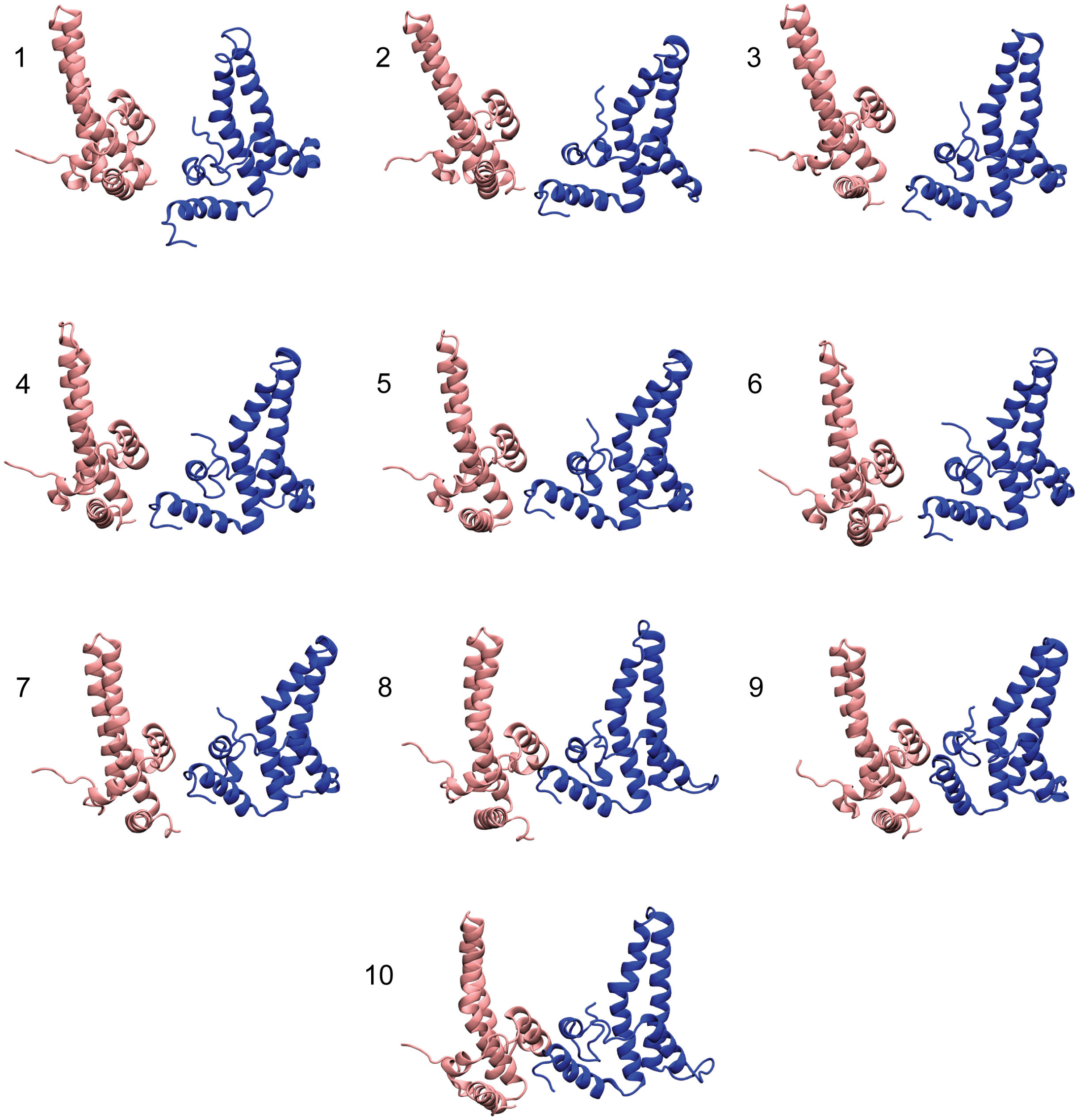

Figure S3: Representative structures along the initial Path 2 selected from targeted molecular dynamics simulations. Images 1–10 correspond to the ten nodes extracted from a single TMD 2 trajectory and used to initialize subsequent string refinement. Structures are shown with a focused view of the inter-dimer gate region involving chains A and F.

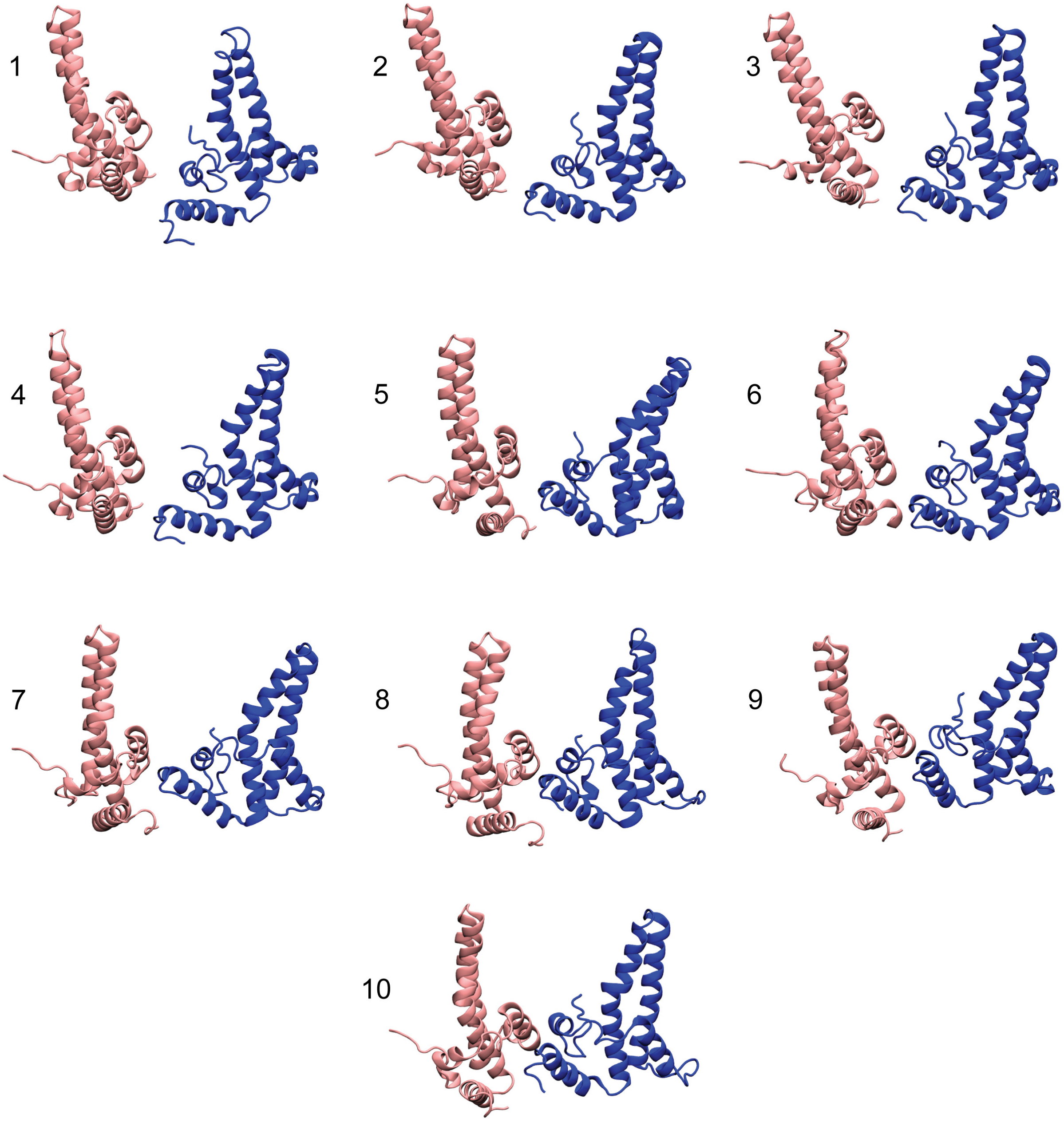

Figure S4: Representative structures along the initial Path 3 selected from targeted molecular dynamics simulations. Images 1–10 correspond to the ten nodes extracted from a single TMD 3 trajectory and used to initialize subsequent string refinement. Structures are shown with a focused view of the inter-dimer gate region involving chains A and F.

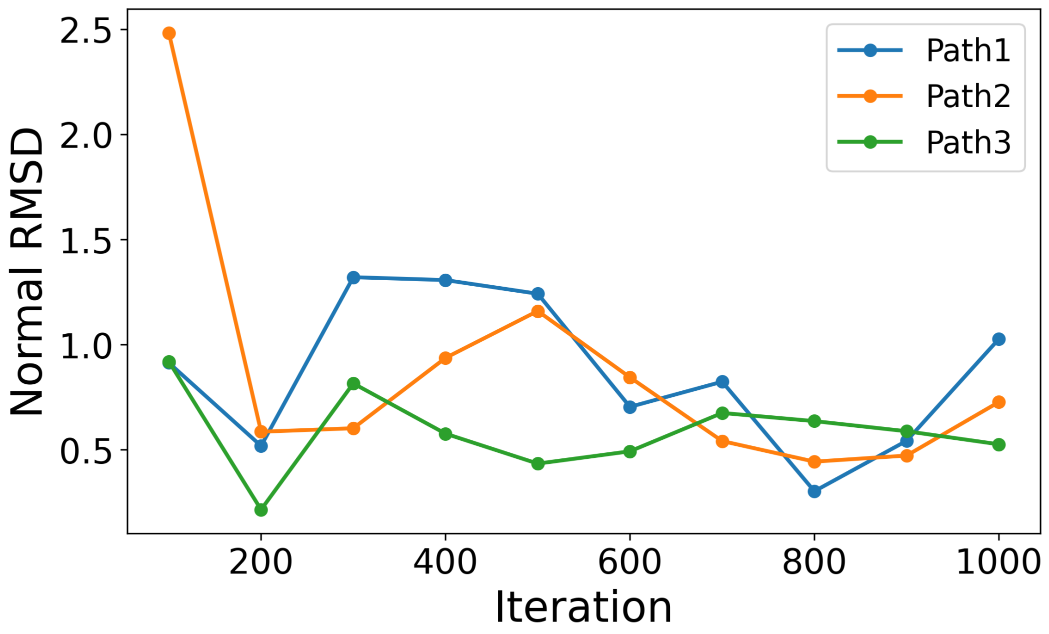

Figure S5: Convergence behavior of string refinement quantified by the normal RMSD between successive saved strings. Normal RMSD is evaluated every 100 iterations and measures displacements perpendicular to the instantaneous string.

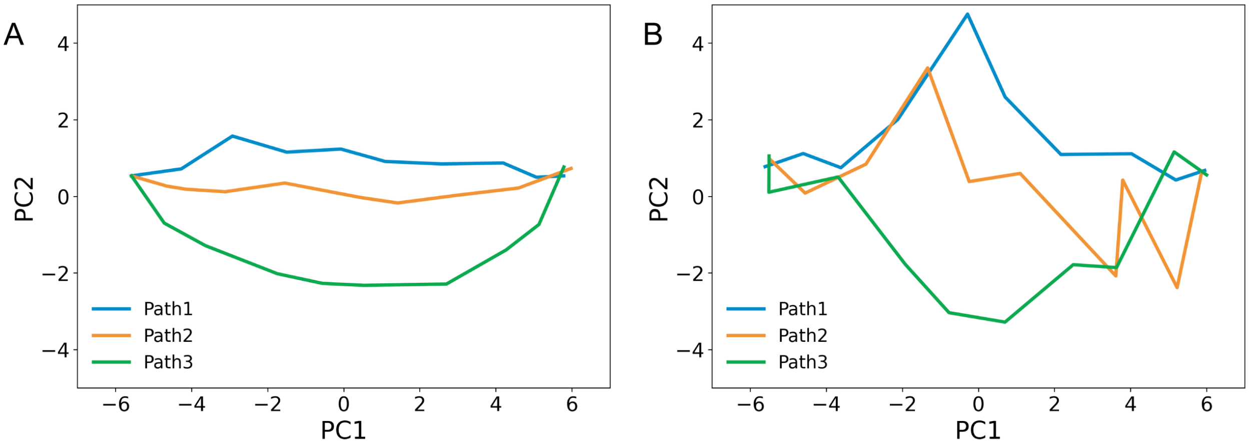

Figure S6: Comparison of initial and refined string pathways in the reduced PC space. **(A)** Overlay of the three initial string paths selected from the TMD trajectories (iteration 0), projected onto the PC1–PC2 plane. **(B)** Overlay of the corresponding refined string paths after 1000 string-method iterations, projected onto the PC1–PC2 plane.

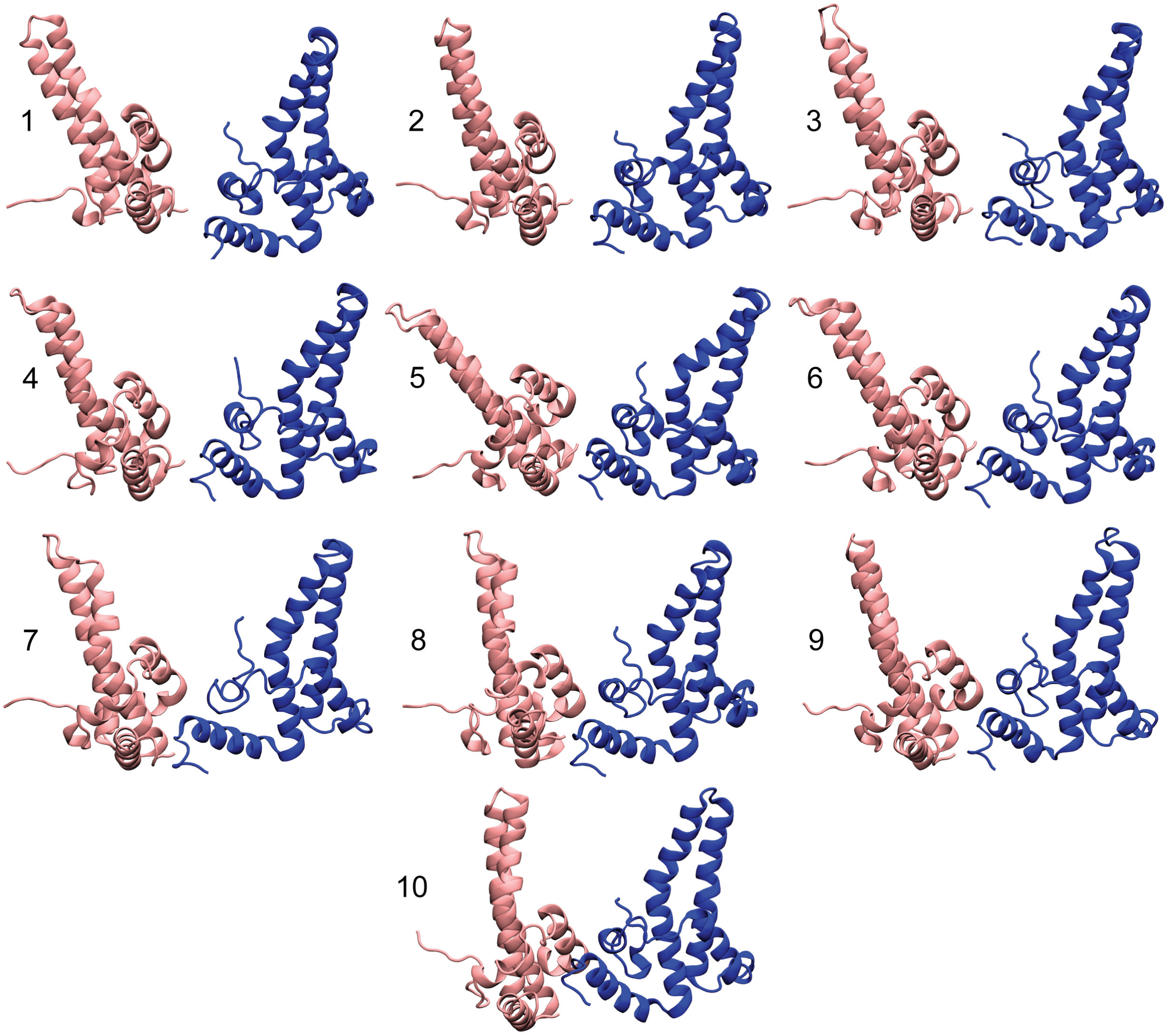

Figure S7: Representative structures along the refined Path 1 after string-method optimization. Images 1–10 correspond to the ten string images obtained after 1000 refinement iterations and represent the final transition pathway used for free-energy sampling. Structures are shown with a focused view of the inter-dimer gate region involving chains A and F.

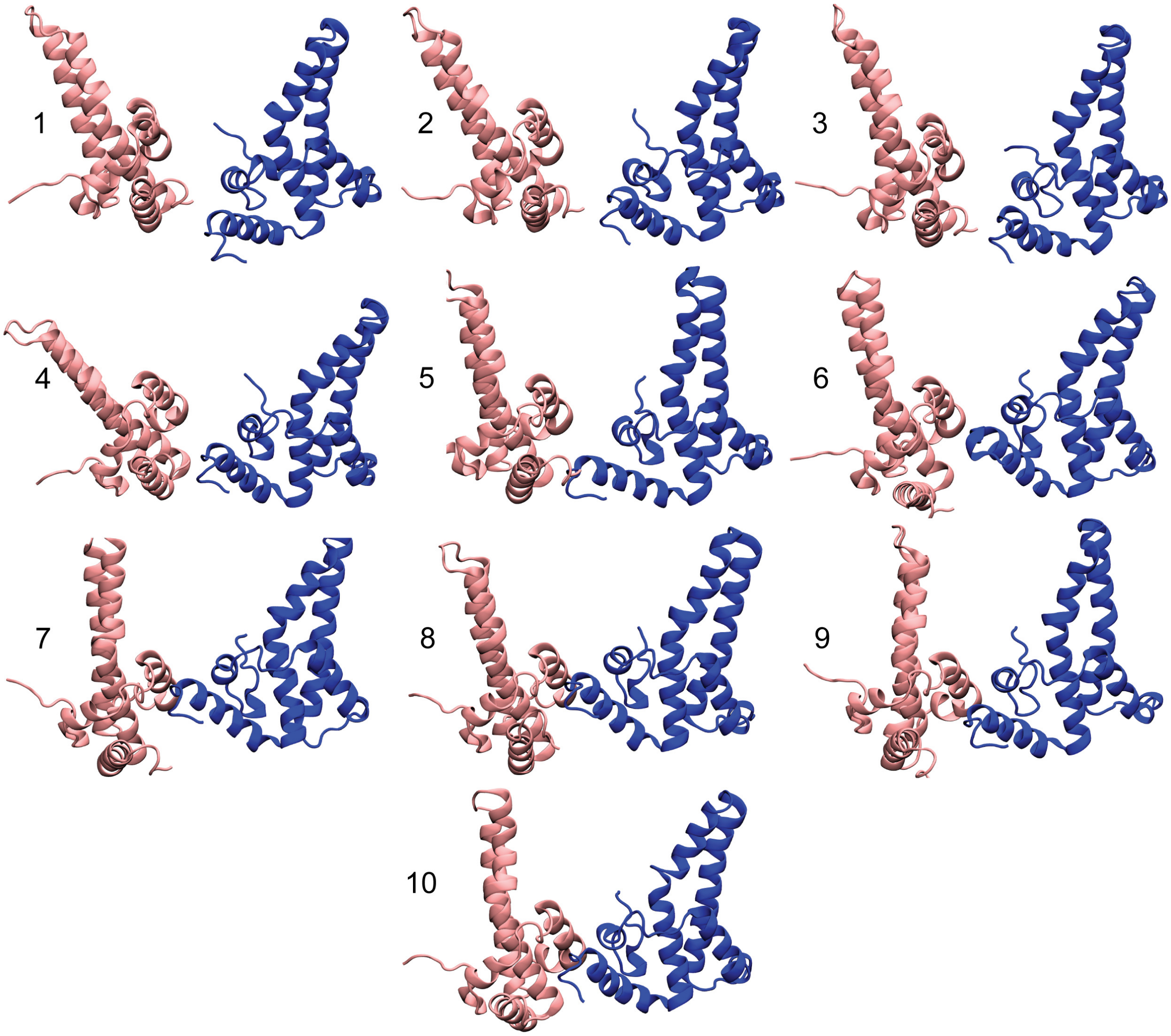

Figure S8: Representative structures along the refined Path 2 after string-method optimization. Images 1–10 correspond to the ten string images obtained after 1000 refinement iterations and represent the final transition pathway used for free-energy sampling. Structures are shown with a focused view of the inter-dimer gate region involving chains A and F.

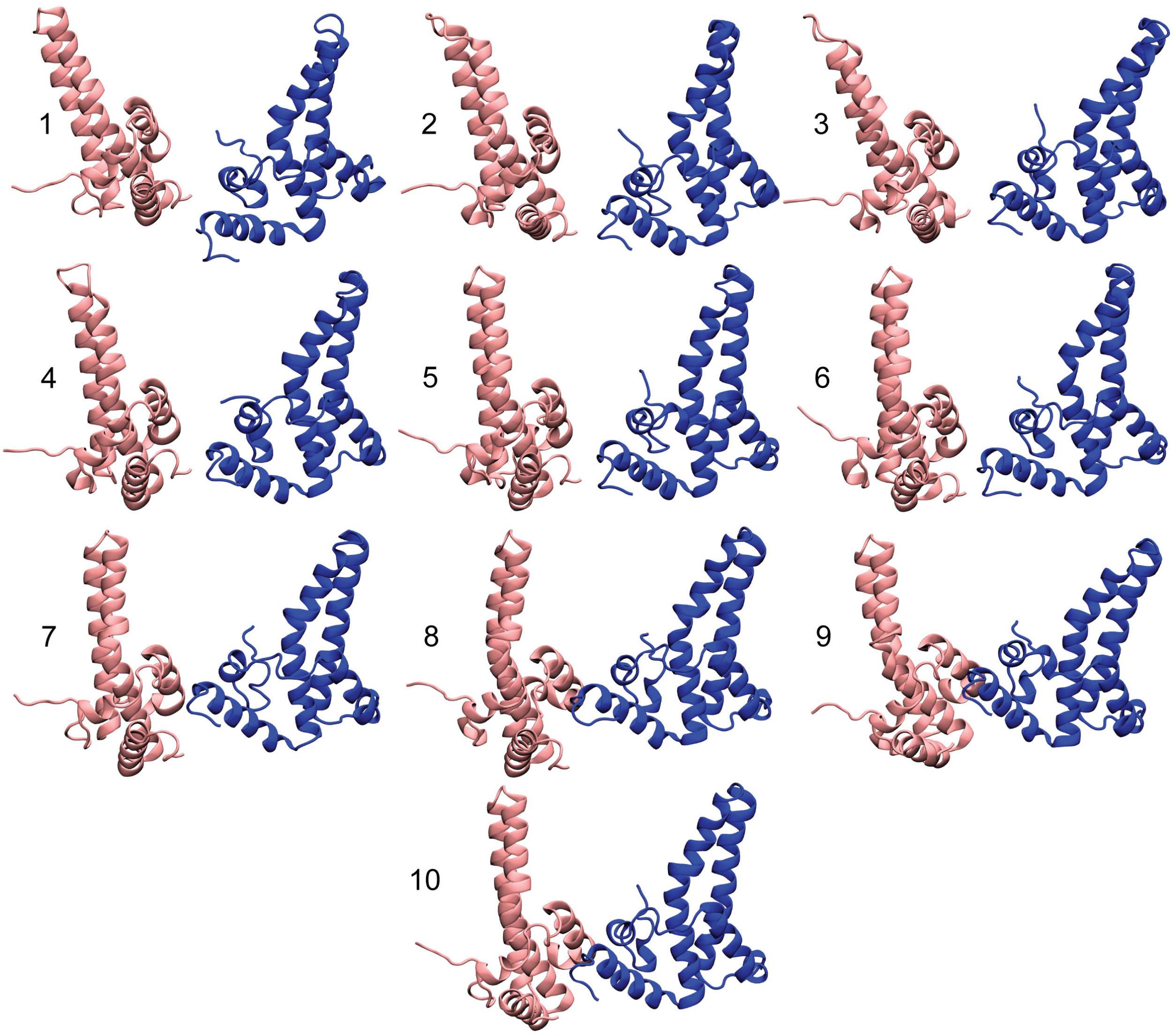

Figure S9: Representative structures along the refined Path 3 after string-method optimization. Images 1–10 correspond to the ten string images obtained after 1000 refinement iterations and represent the final transition pathway used for free-energy sampling. Structures are shown with a focused view of the inter-dimer gate region involving chains A and F.

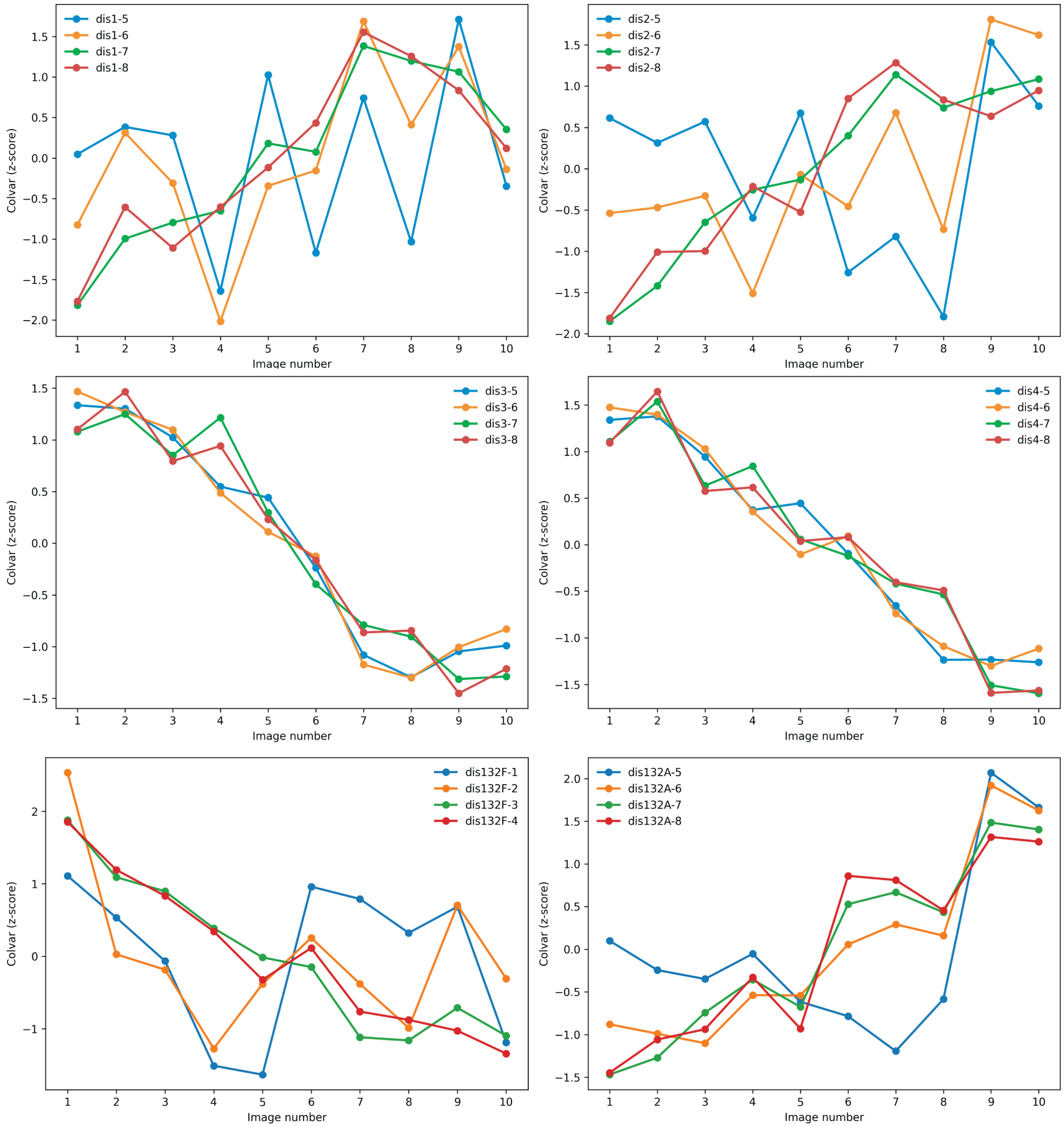

Figure S10: Z-scores (mean-centered and scaled by the standard deviation) of distance-based collective variables, as defined in Table S1, plotted versus image number (1–10) for Path 1 at iteration 1000. Based on smooth, systematic variation along the refined paths, the following 16 collective variables were retained for construction of the pathCV distance metric: dis1-7, dis2-7, dis2-8, dis3-5, dis3-6, dis3-7, dis3-8, dis4-5, dis4-6, dis4-7, dis4-8, dis132F-3, dis132F-4, dis132A-6, dis132A-7, and dis132A-8. Collective variables not listed were excluded due to noisy variation along the path.

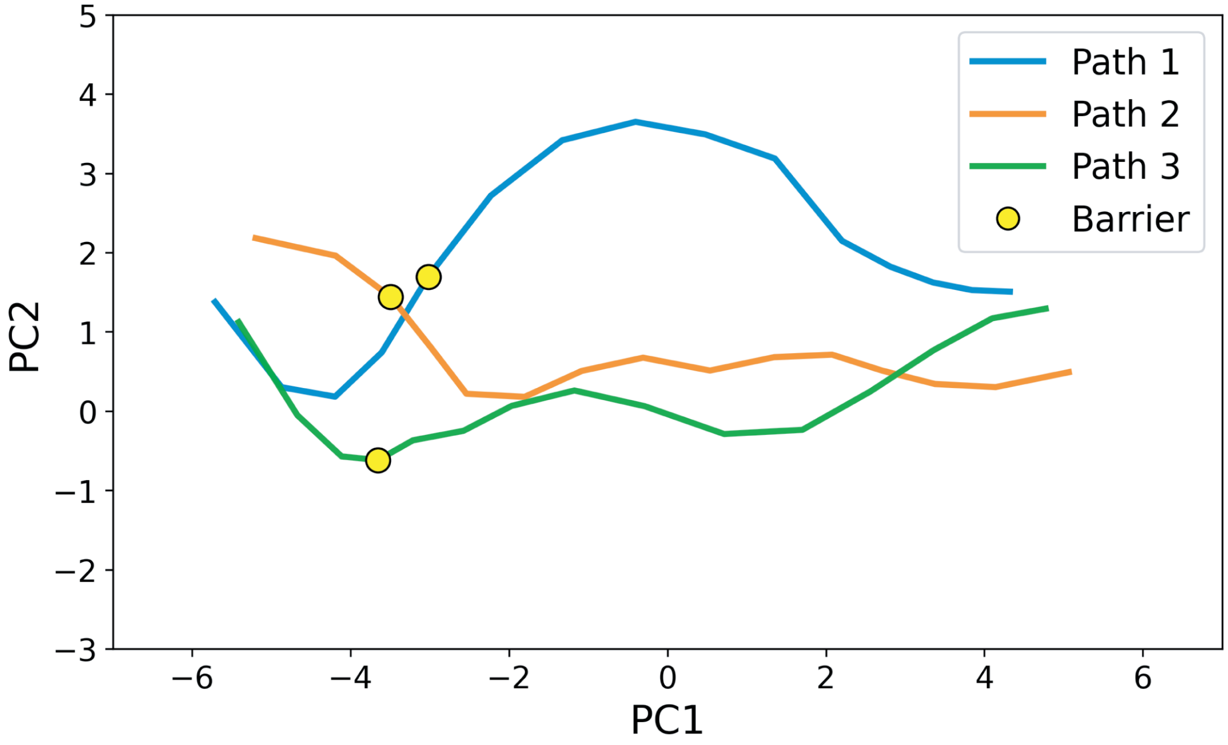

Figure S11: Realized minimum free-energy pathways projected onto the reduced PC space. The minimum free-energy paths (MFEPs) corresponding to the three refined transition pathways are overlaid in the PC1–PC2 plane, with the locations of the rate-limiting configurations marked by yellow circles.

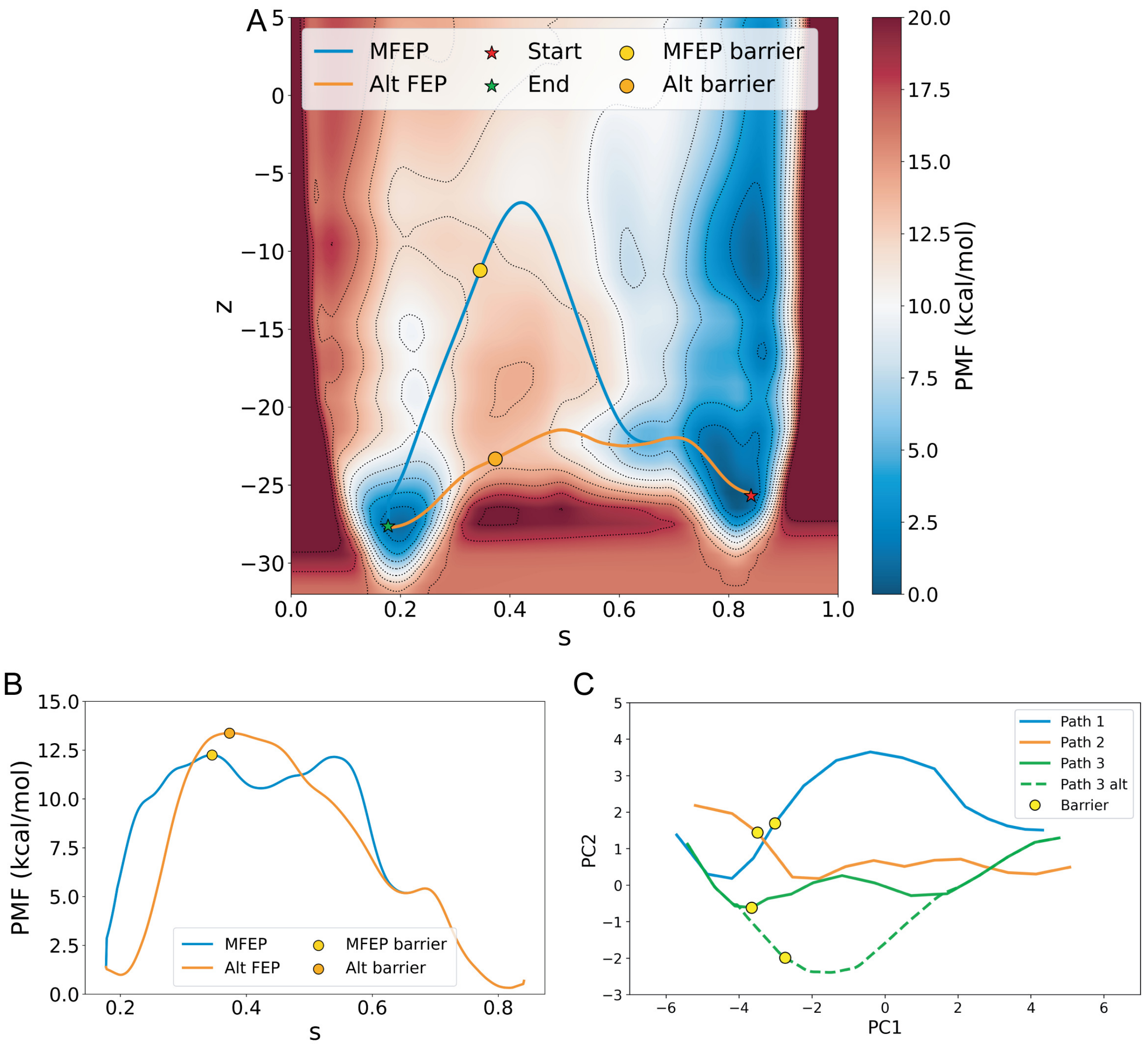

Figure S12: Comparison of the optimal and constrained alternative transition pathways for Path 3. **(A)** Two–dimensional free–energy surface in the (s, z) path collective variables for Path 3, with the extracted MFEP and an alternative path constrained to lower z values overlaid. The locations of the corresponding free–energy barriers along each path are indicated by circles. **(B)** One–dimensional free–energy profiles along the MFEP and the alternative path. The barrier positions along each profile are marked by circles. **(C)** Projection of the realized molecular pathways onto the PC1–PC2 space, showing the three MFEPs together with the alternative Path 3 realization.

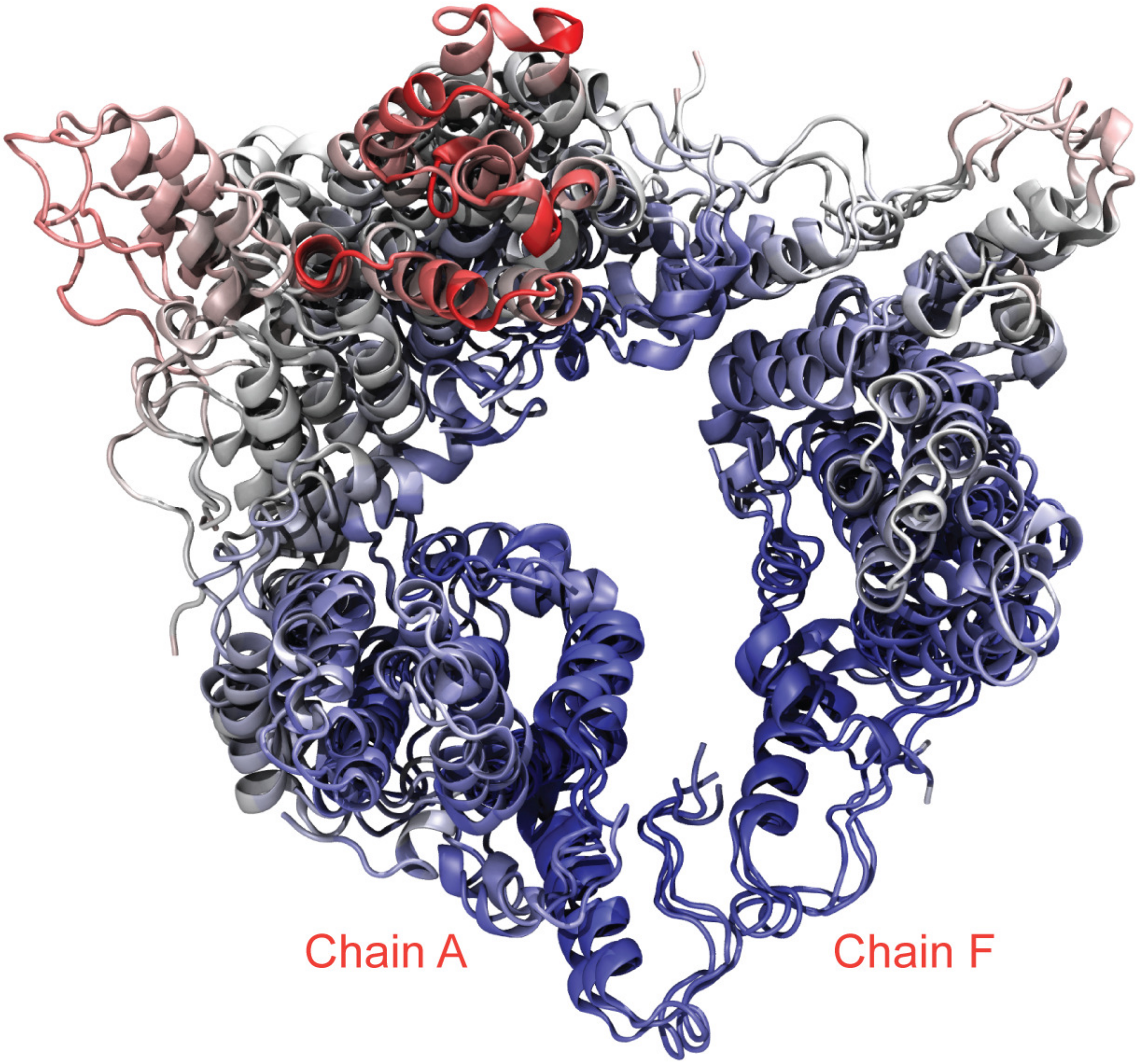

Figure S13: Overlay of the complete rate-limiting conformations extracted from the three realized transition pathways. The structures show good agreement in the local gate region that defines the dominant free-energy barrier, while noticeable variability remains in other parts of the hexamer. Coloring indicates per-residue structural dispersion across the three aligned conformations, with blue denoting regions of high structural agreement and red denoting increased pathway-to-pathway variability. These differences reflect additional degrees of freedom not explicitly captured by the collective variables used for path definition and free-energy sampling, and likely contribute to the residual variability observed in the numerical free-energy estimates.

Table S1: Definition of atomic groups (C_α selections) used to construct the distance-based collective variables in the gate region. Groups are defined by the residue ranges used in the VMD atom selections. A structural visualization of the selected groups is shown in Fig. 2.

**Group**	**Chain**	**Residues**
1	A	112–119
2	A	120–128
3	A	27–32
4	A	33–38
5	F	112–119
6	F	120–127
7	F	13–15
8	F	16–19
Tyr132A	A	132
Tyr132F	F	132

Table S2: Definition of the 24 distance-based collective variables (colvars) constructed from the atomic groups listed in Table S1. Each collective variable, denoted as dis$i$–j, corresponds to the distance between the centers of mass (computed over C_α atoms only) of atomic Group i and Group j as defined in Table S1.

**Colvar**	**Group i**	**Group j**
dis1–5	1	5
dis1–6	1	6
dis1–7	1	7
dis1–8	1	8
dis2–5	2	5
dis2–6	2	6
dis2–7	2	7
dis2–8	2	8
dis3–5	3	5
dis3–6	3	6
dis3–7	3	7
dis3–8	3	8
dis4–5	4	5
dis4–6	4	6
dis4–7	4	7
dis4–8	4	8
dis132–1	Tyr132F	1
dis132–2	Tyr132F	2
dis132–3	Tyr132F	3
dis132–4	Tyr132F	4
dis132–5	Tyr132A	5
dis132–6	Tyr132A	6
dis132–7	Tyr132A	7
dis132–8	Tyr132A	8

Table S3: Loadings of the 24 distance-based collective variables (colvars) on the first two principal components (PC1 and PC2) obtained from PCA of the pooled TMD trajectories.

**Colvar**	**PC1 loading**	**PC2 loading**
dis1–5	0.0913	0.1980
dis1–6	-0.0355	-0.0627
dis1–7	-0.1971	-0.0795
dis1–8	-0.1495	-0.1453
dis2–5	0.0690	0.5610
dis2–6	-0.0952	0.3520
dis2–7	-0.2190	0.0007
dis2–8	-0.1884	-0.0401
dis3–5	0.2759	0.0570
dis3–6	0.2724	0.0709
dis3–7	0.2520	-0.0059
dis3–8	0.2570	-0.0041
dis4–5	0.2782	-0.0063
dis4–6	0.2647	-0.0237
dis4–7	0.2304	-0.0763
dis4–8	0.2390	-0.0812
dis132F–1	0.0935	-0.1459
dis132F–2	0.1059	0.3774
dis132F–3	0.2640	0.0969
dis132F–4	0.2533	-0.0081
dis132A–5	-0.0912	0.4698
dis132A–6	-0.1860	0.2672
dis132A–7	-0.2271	0.0463
dis132A–8	-0.2152	0.0213

Table S4: Free-energy characteristics of the minimum free-energy paths (MFEPs) obtained from the three pathCV landscapes. Energies are reported in kcal/mol. F_{start} and F_{end} correspond to the open and closed basins, respectively. F_{peak} is the absolute free energy at the highest point along the MFEP, $F^{\ddagger}$ is the barrier height relative to the open state, and $\Delta F = F_{\text{end}} - F_{\text{start}}$.

**Path**	$\boldsymbol{F_{\text{start}}}$	$\boldsymbol{F_{\text{peak}}}$	$\boldsymbol{F_{\text{end}}}$	$\boldsymbol{F^{\ddagger}}$	$\boldsymbol{\Delta F}$
1	4.52	13.61	0.28	9.10	-4.24
2	3.20	10.66	0.71	7.46	-2.49
3	1.39	12.25	0.32	10.86	-1.07
Mean ± SD	–	–	–	9.14 ± 1.70	-2.59 ± 1.60
